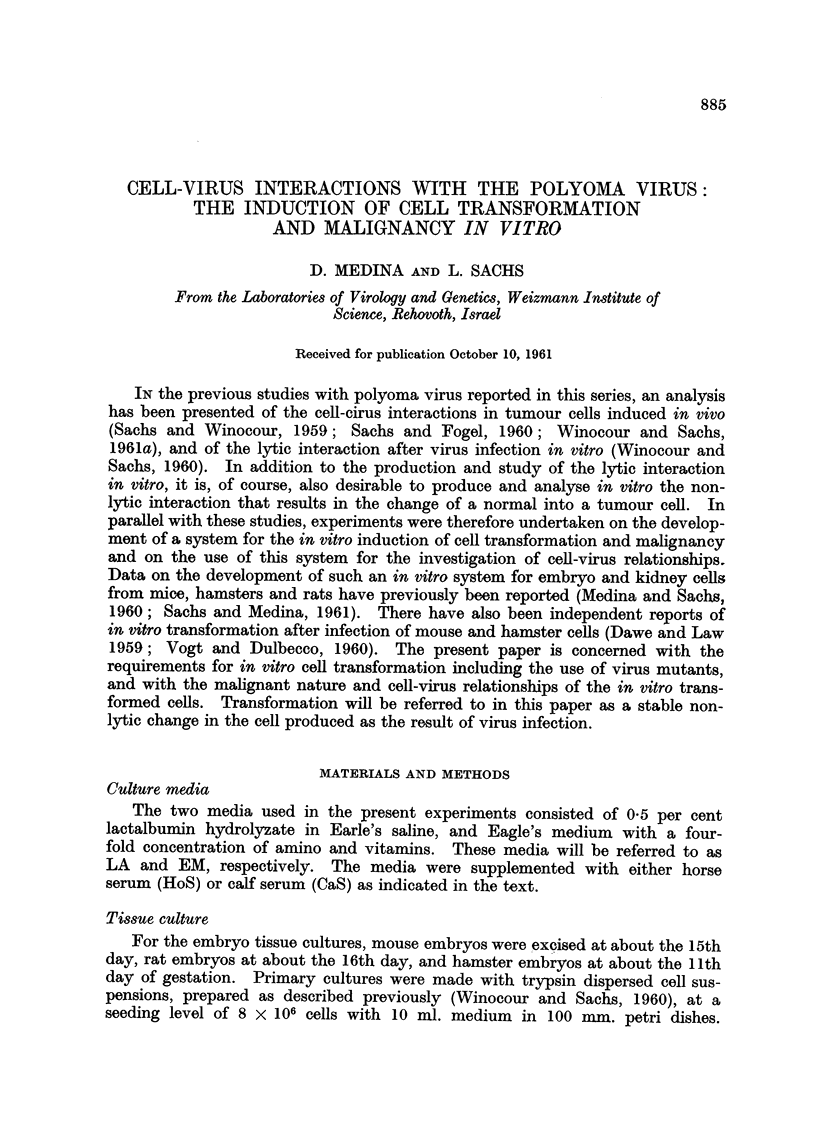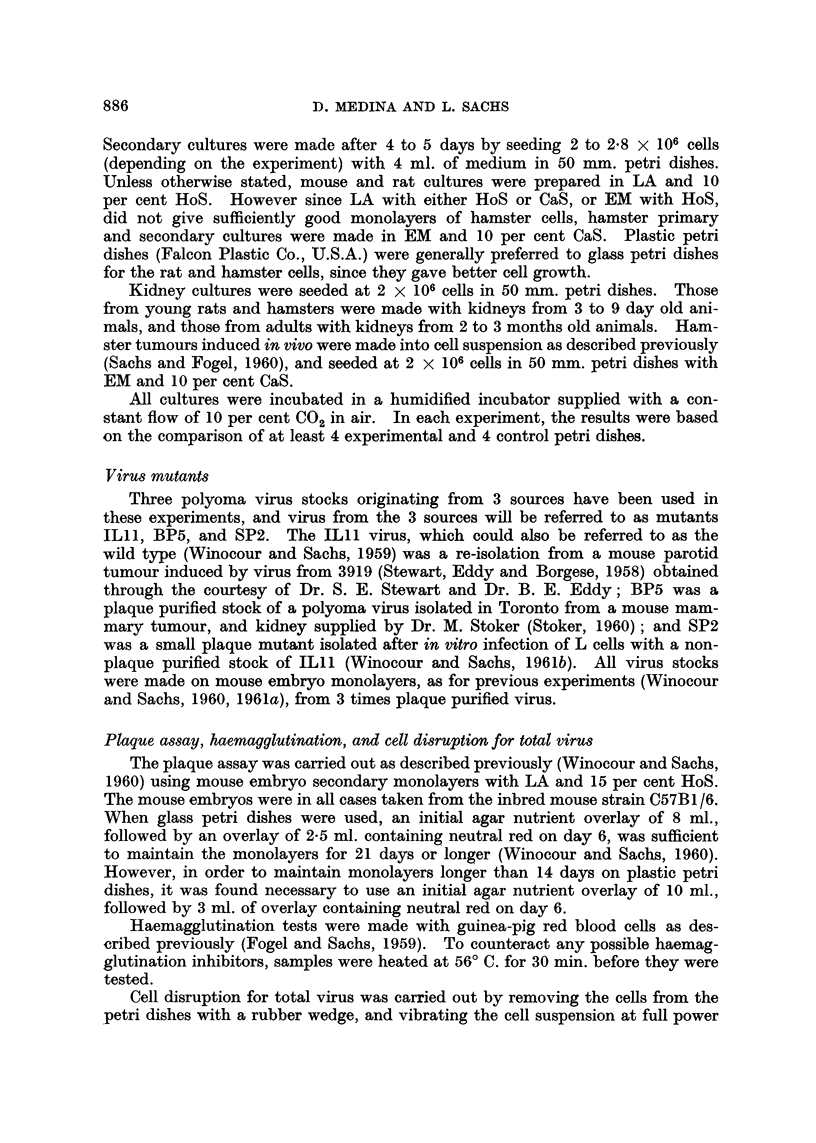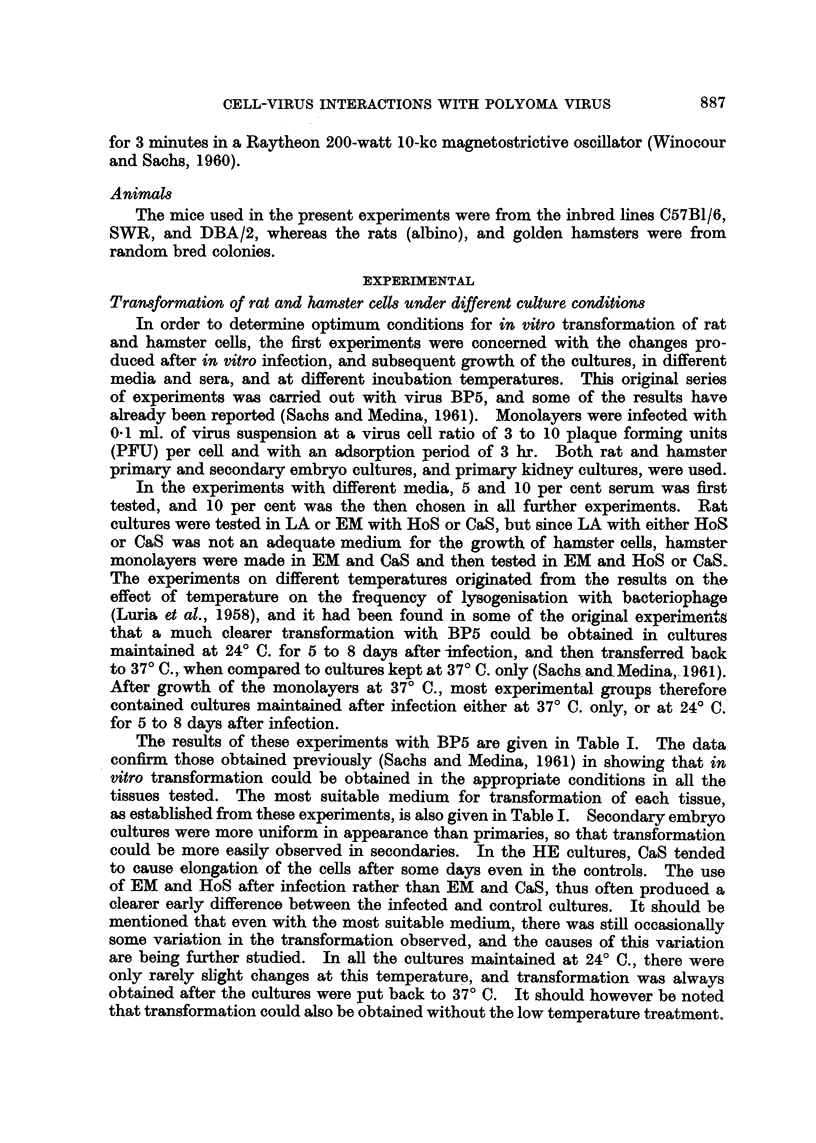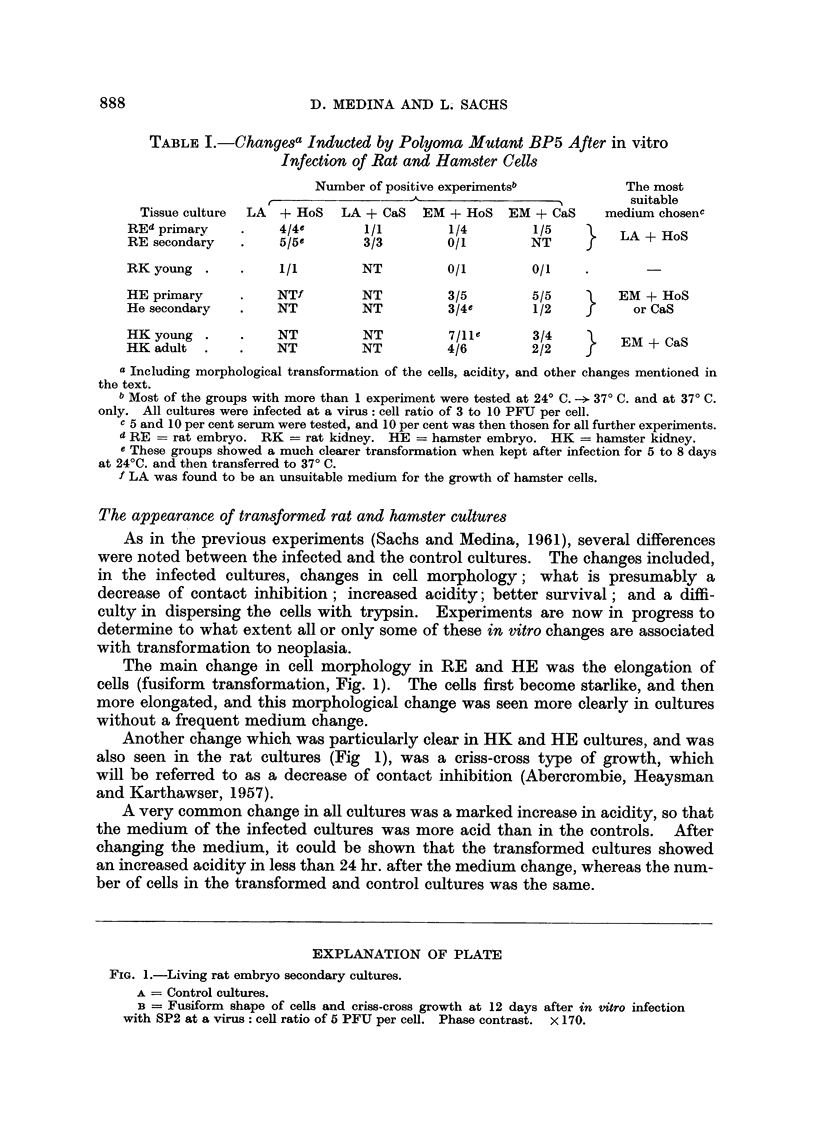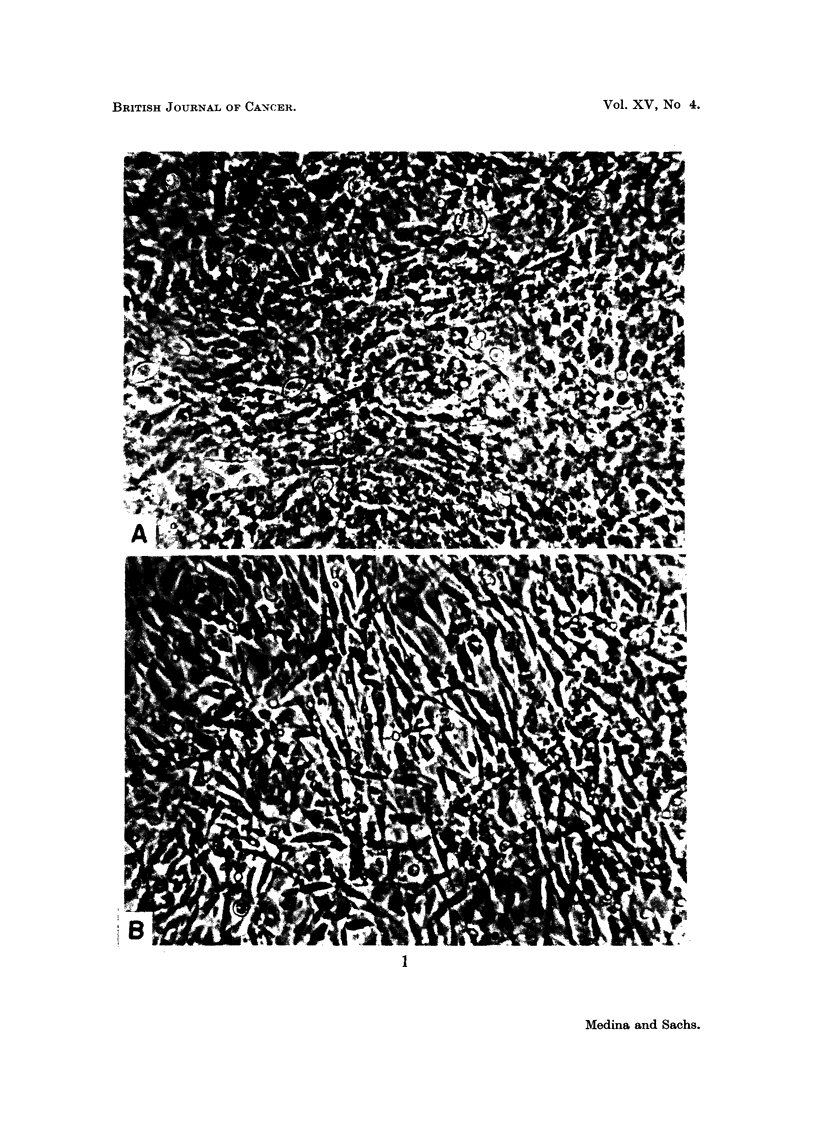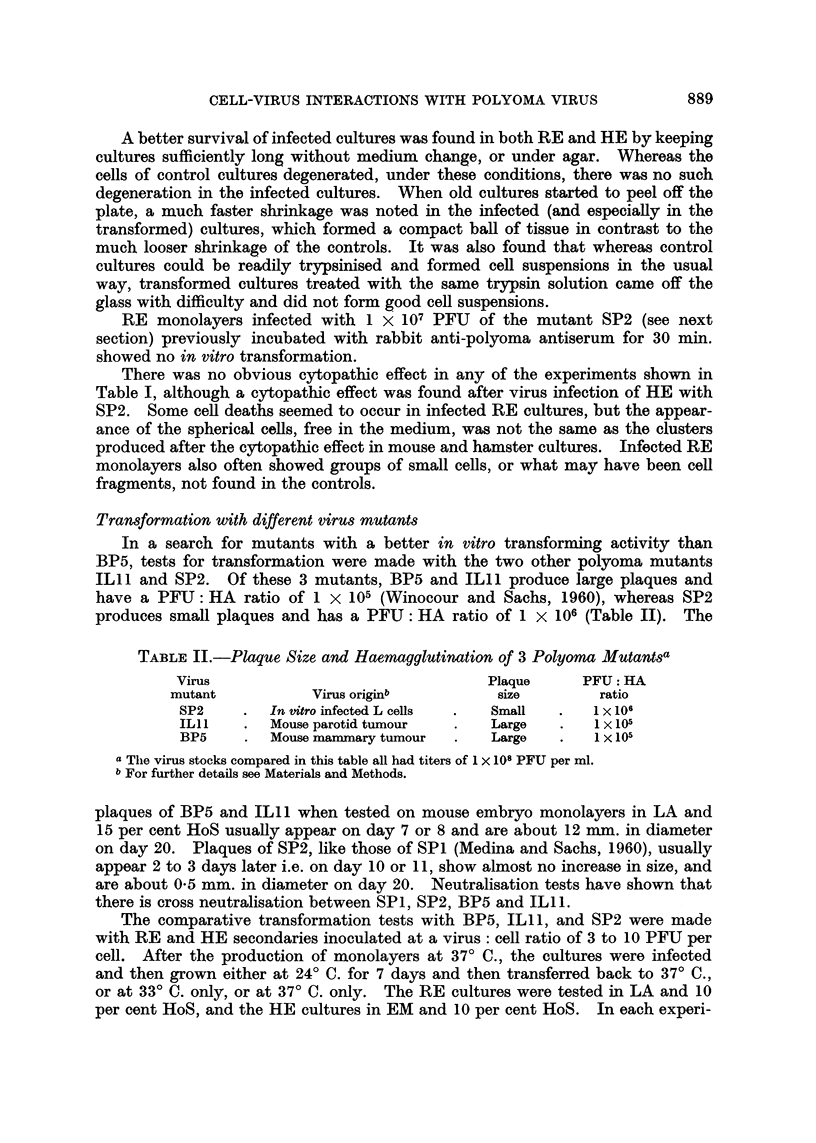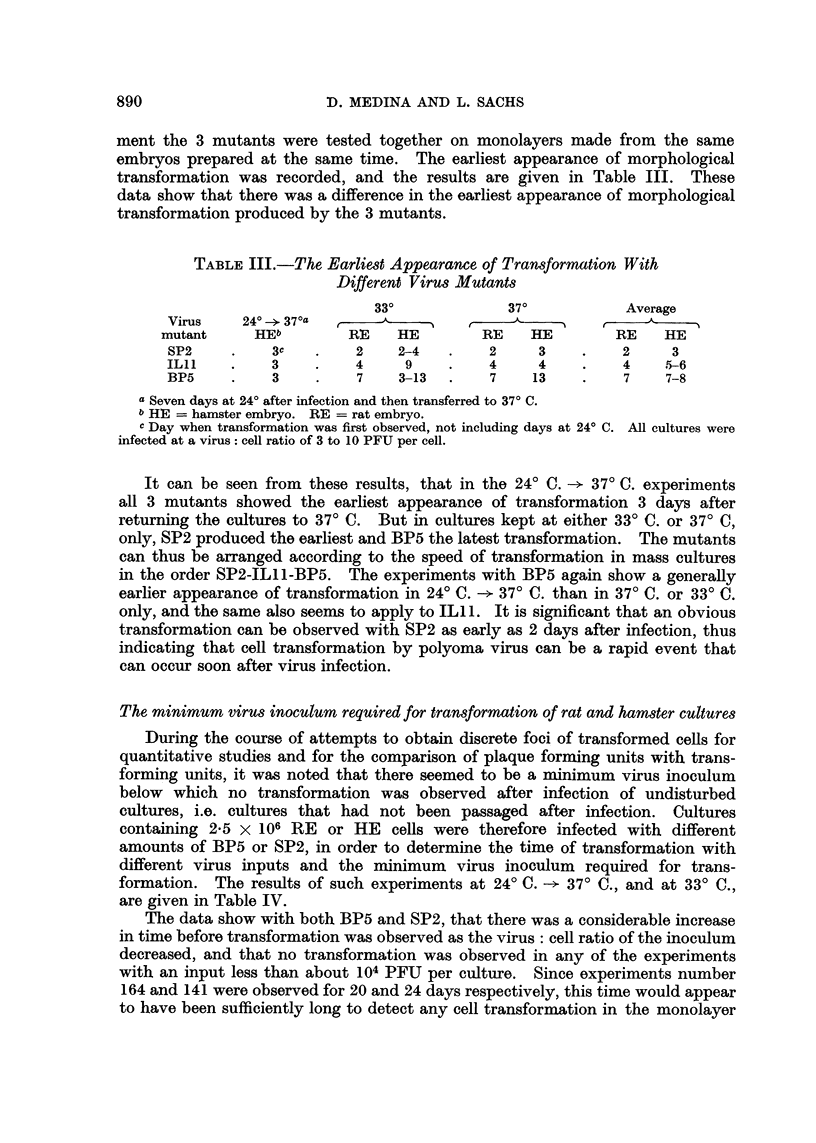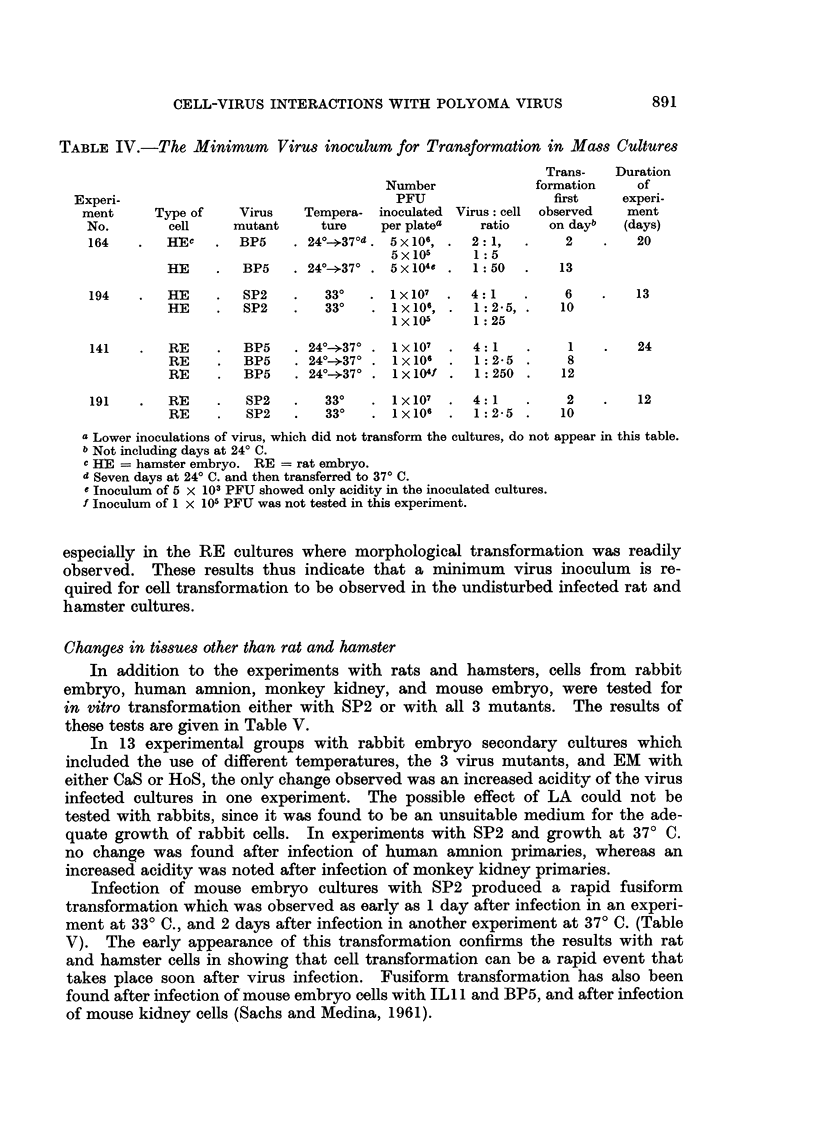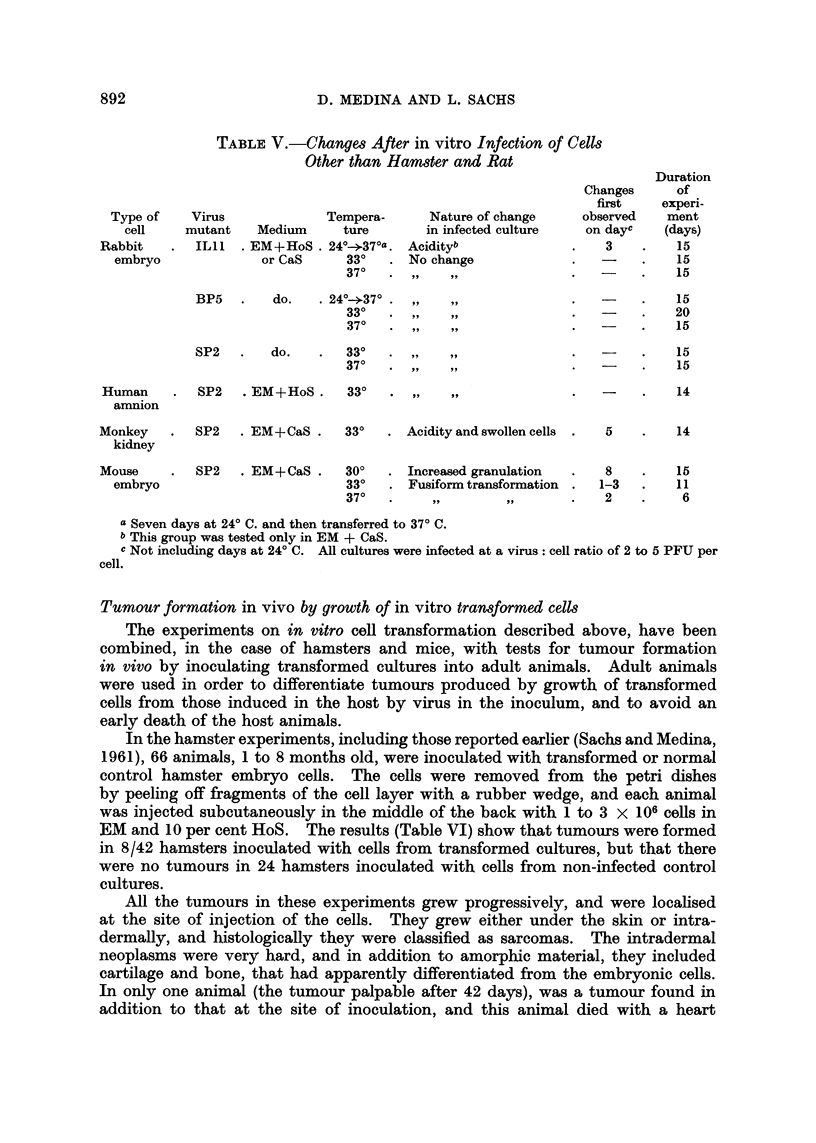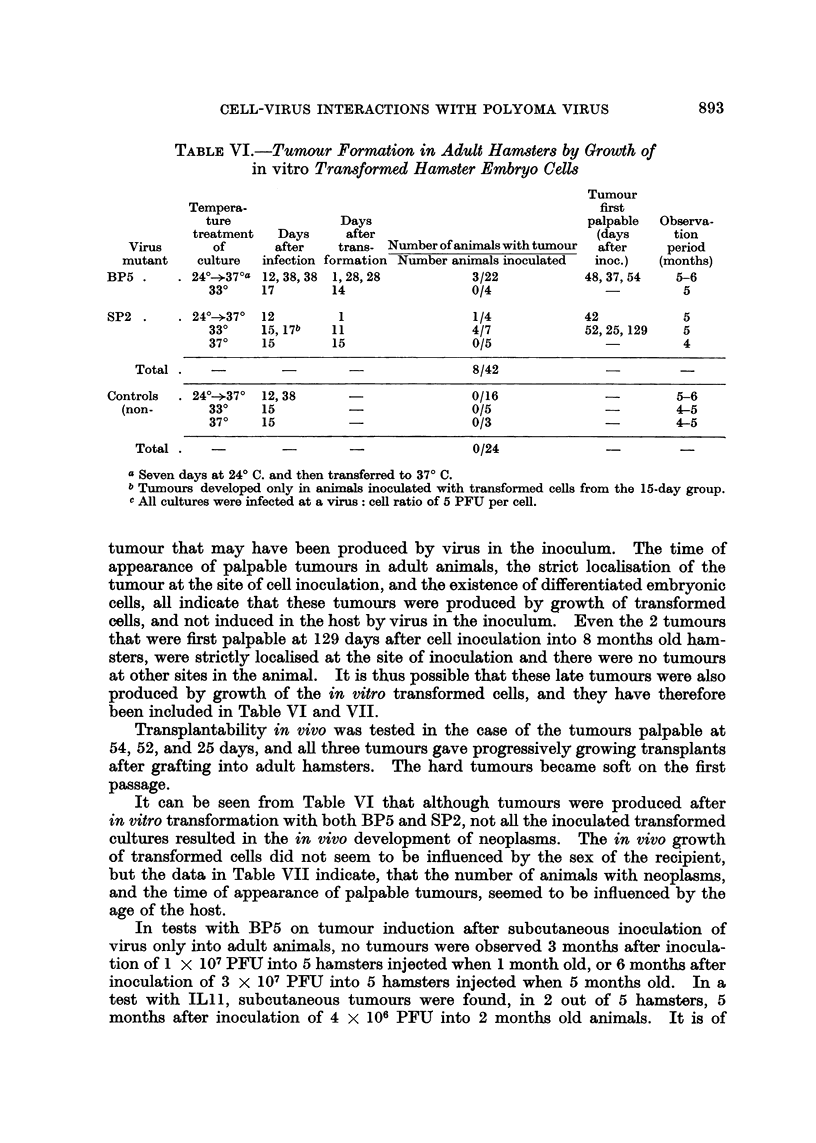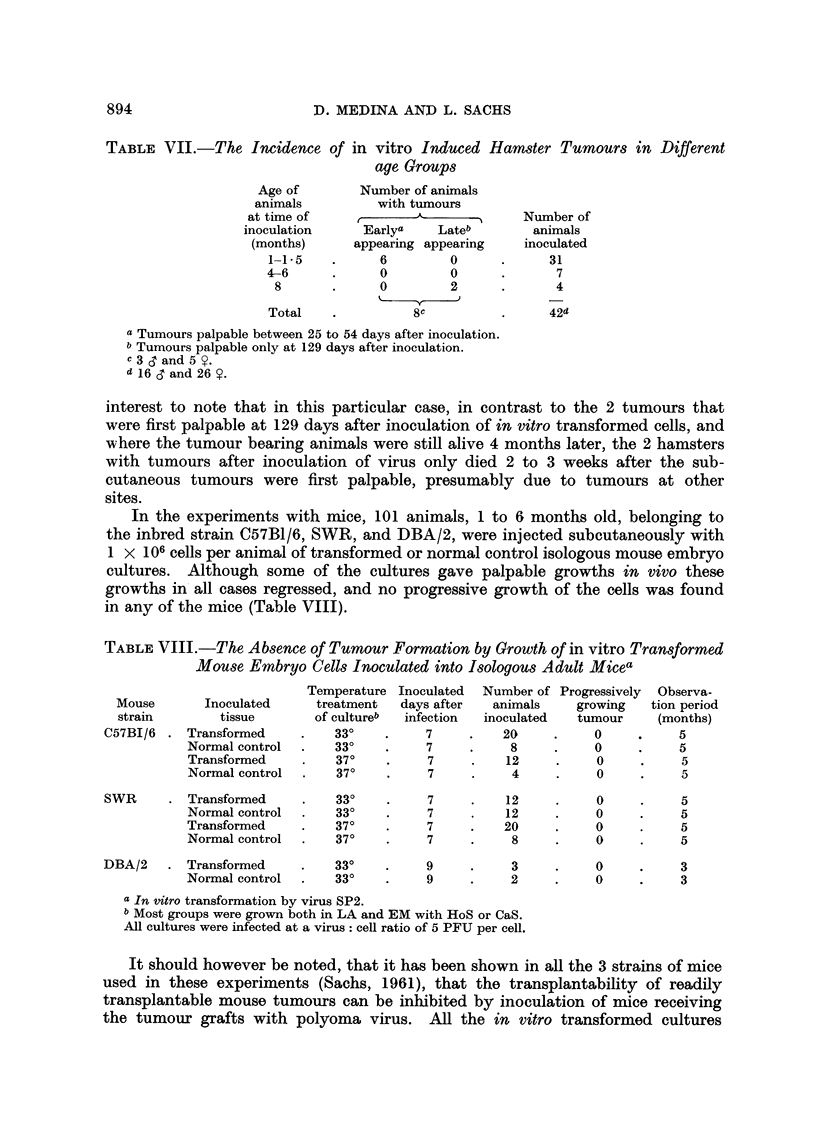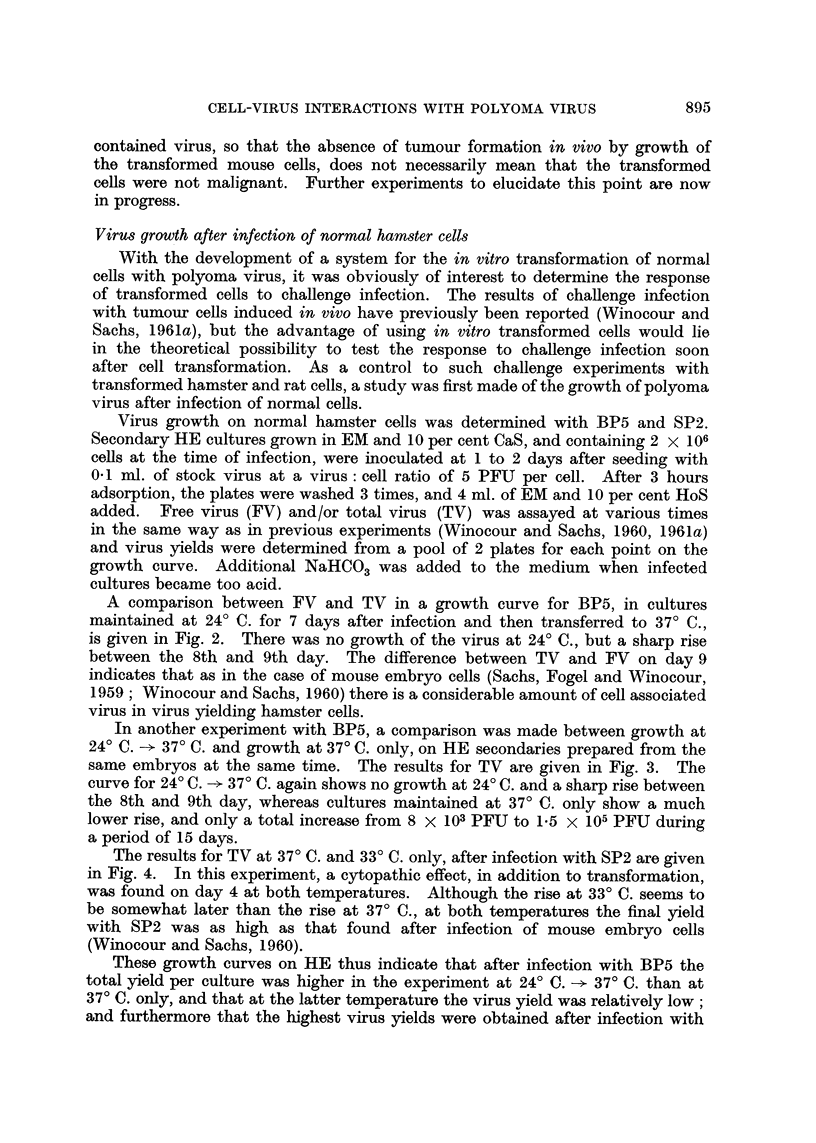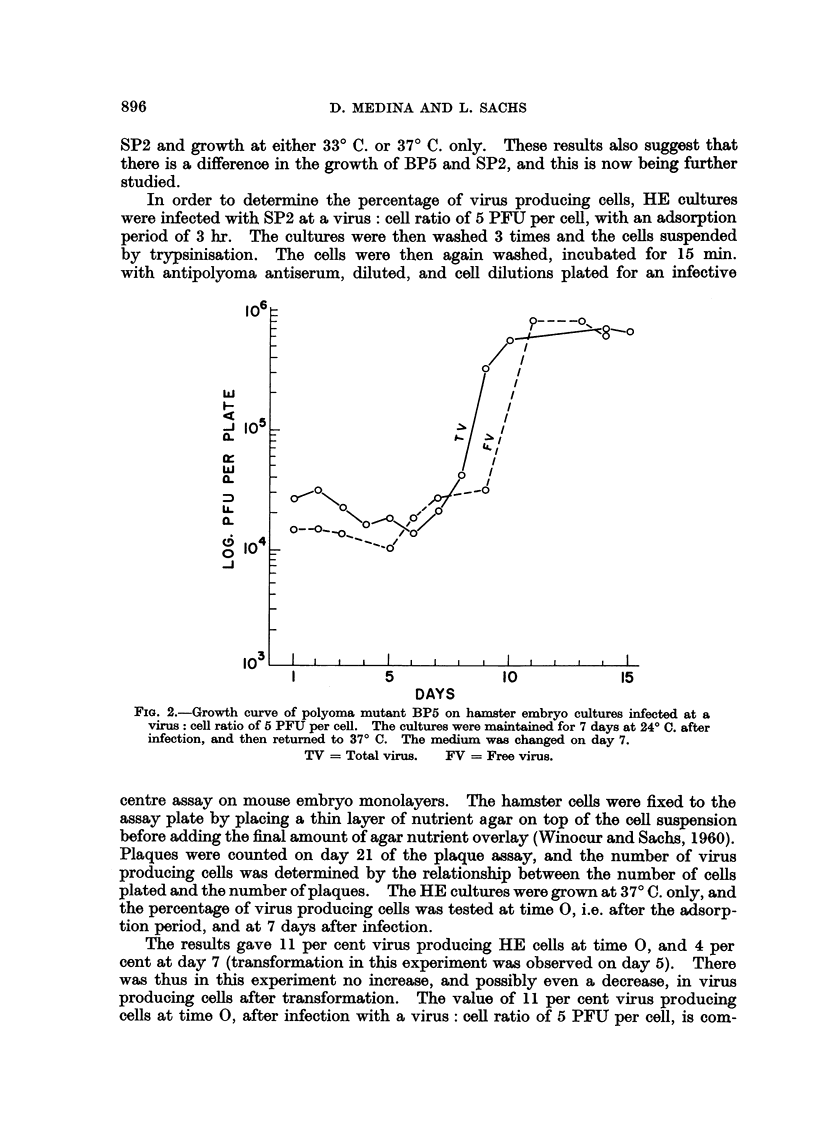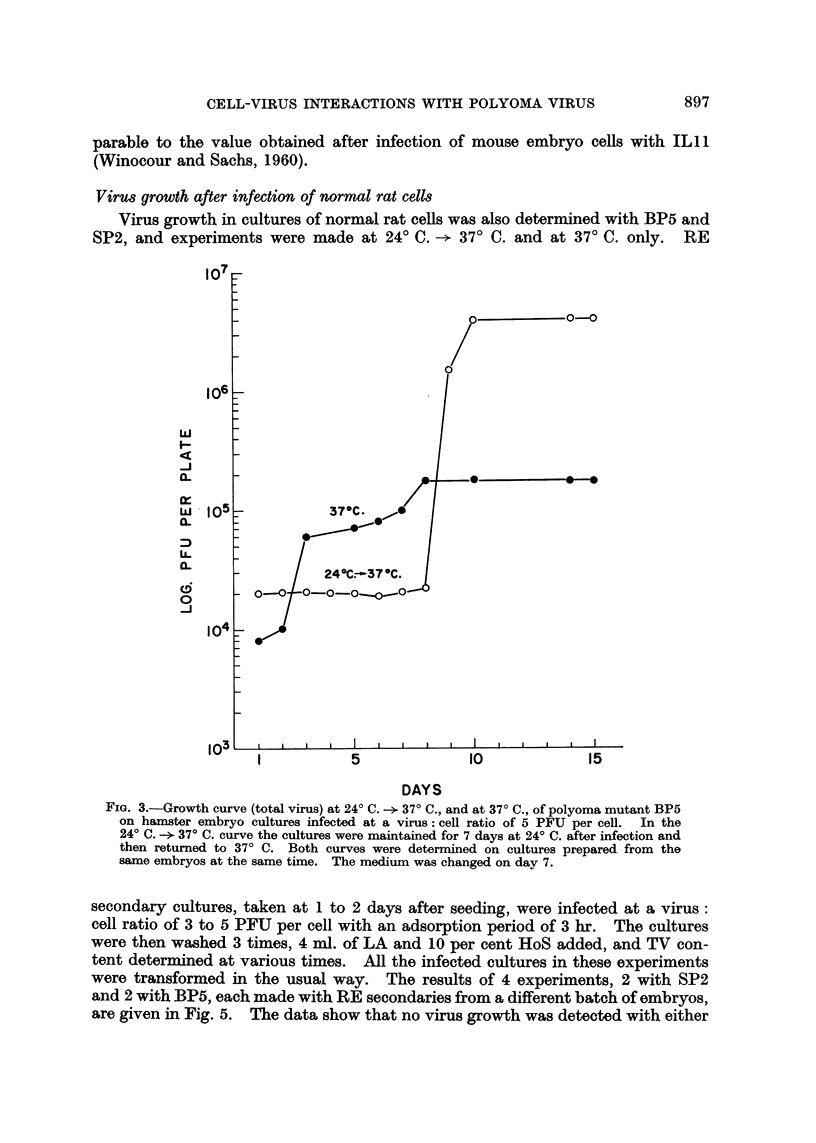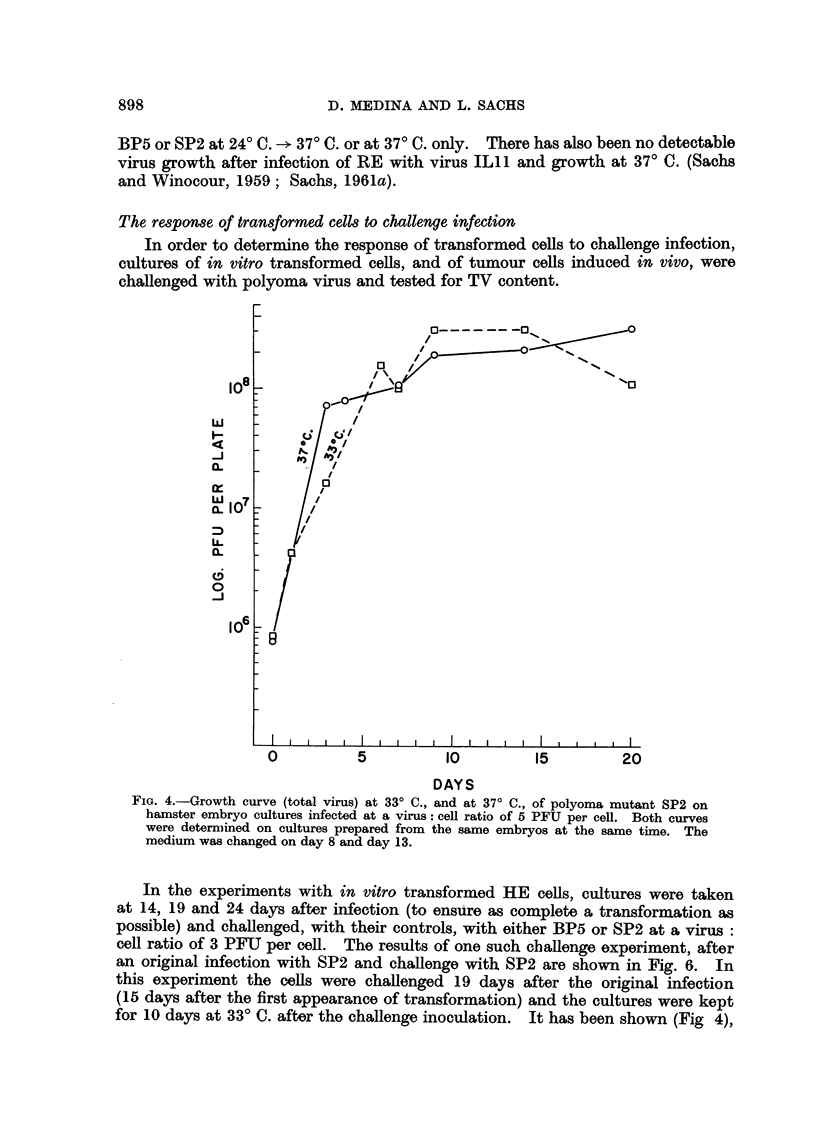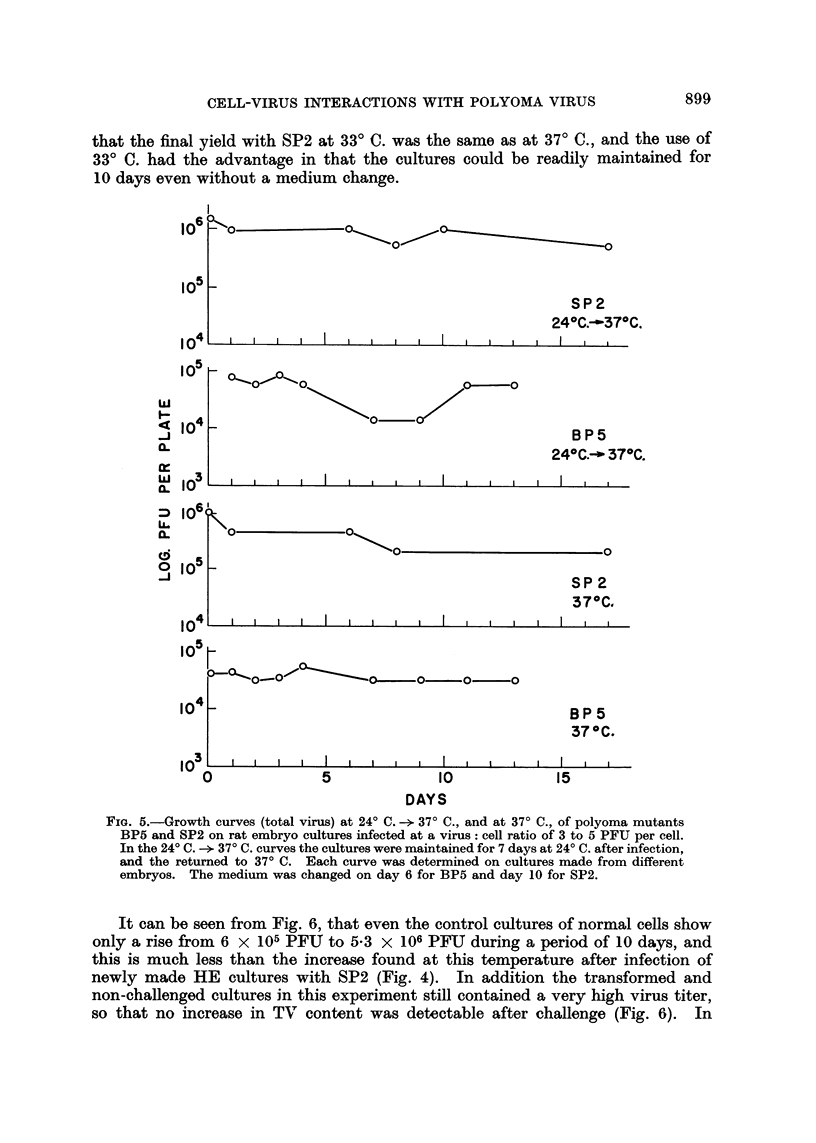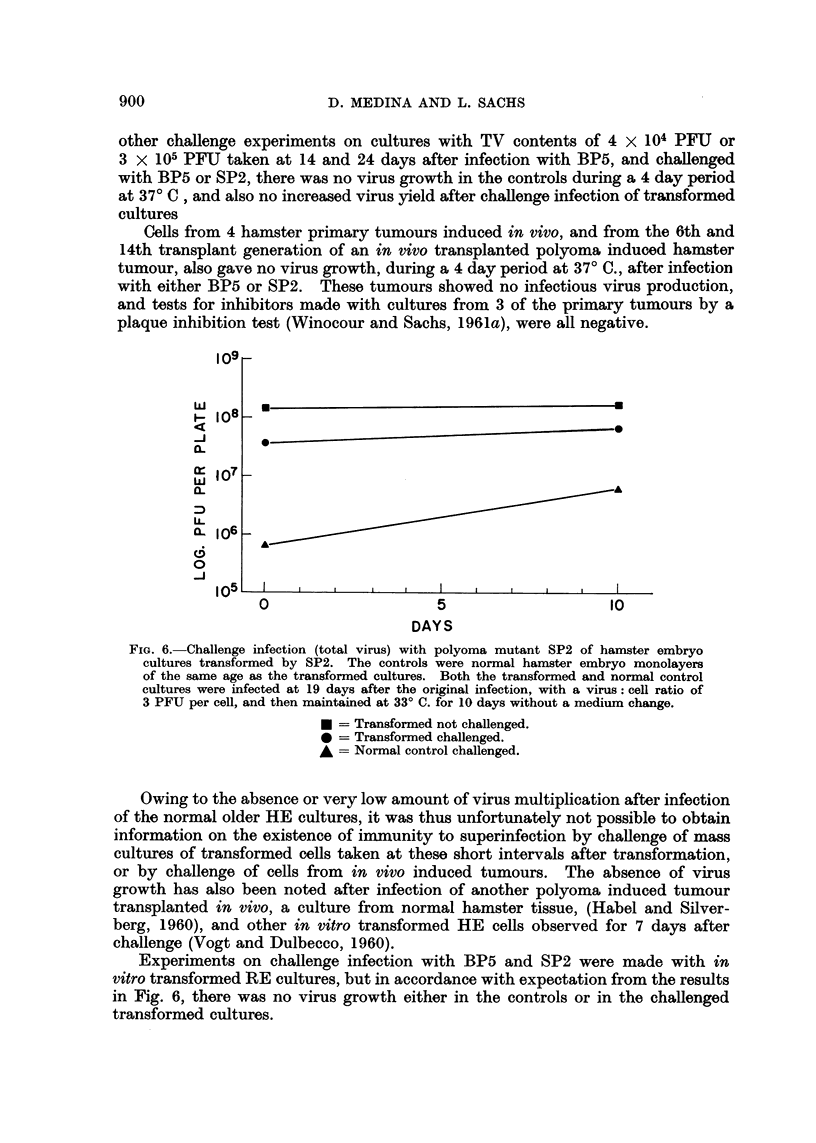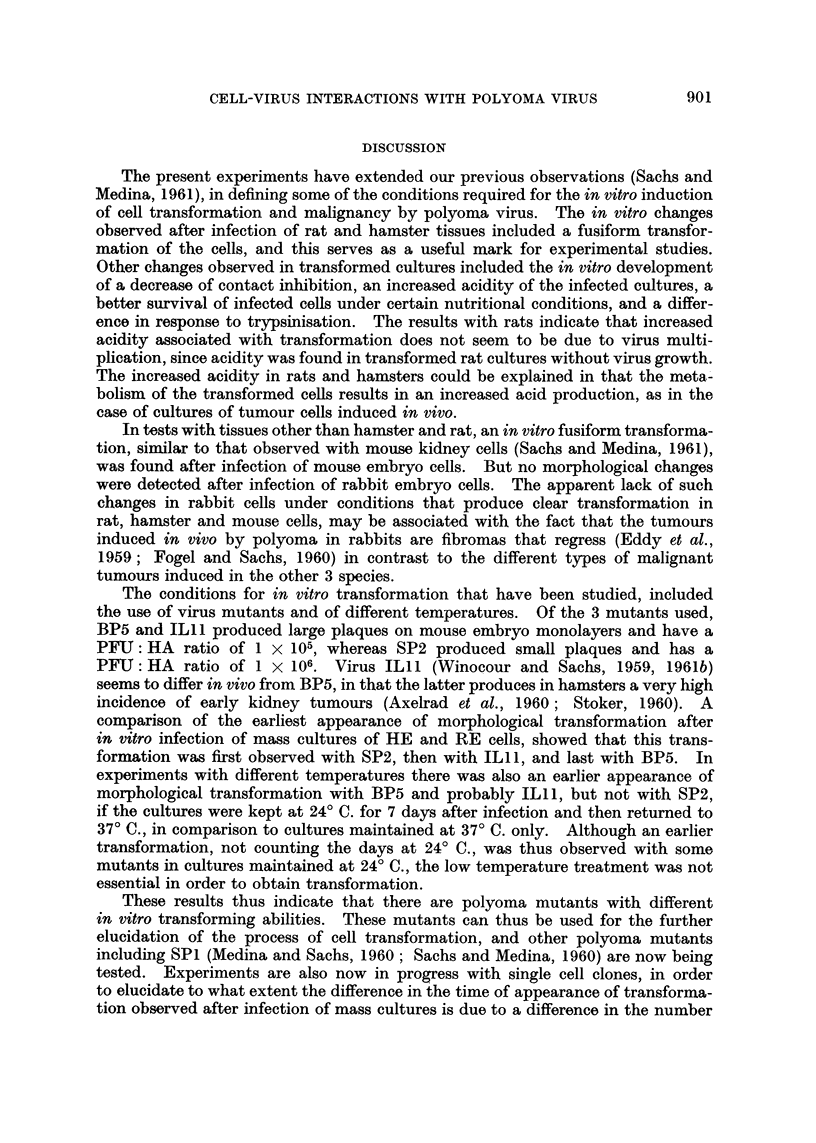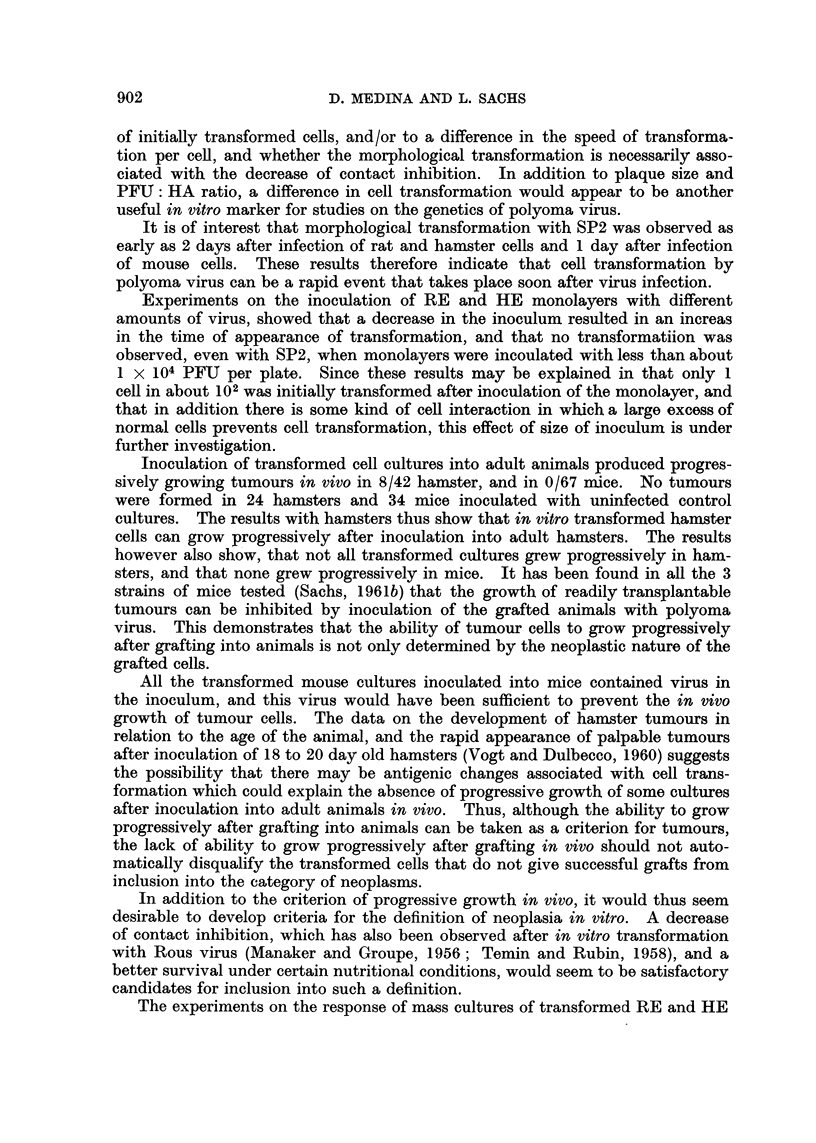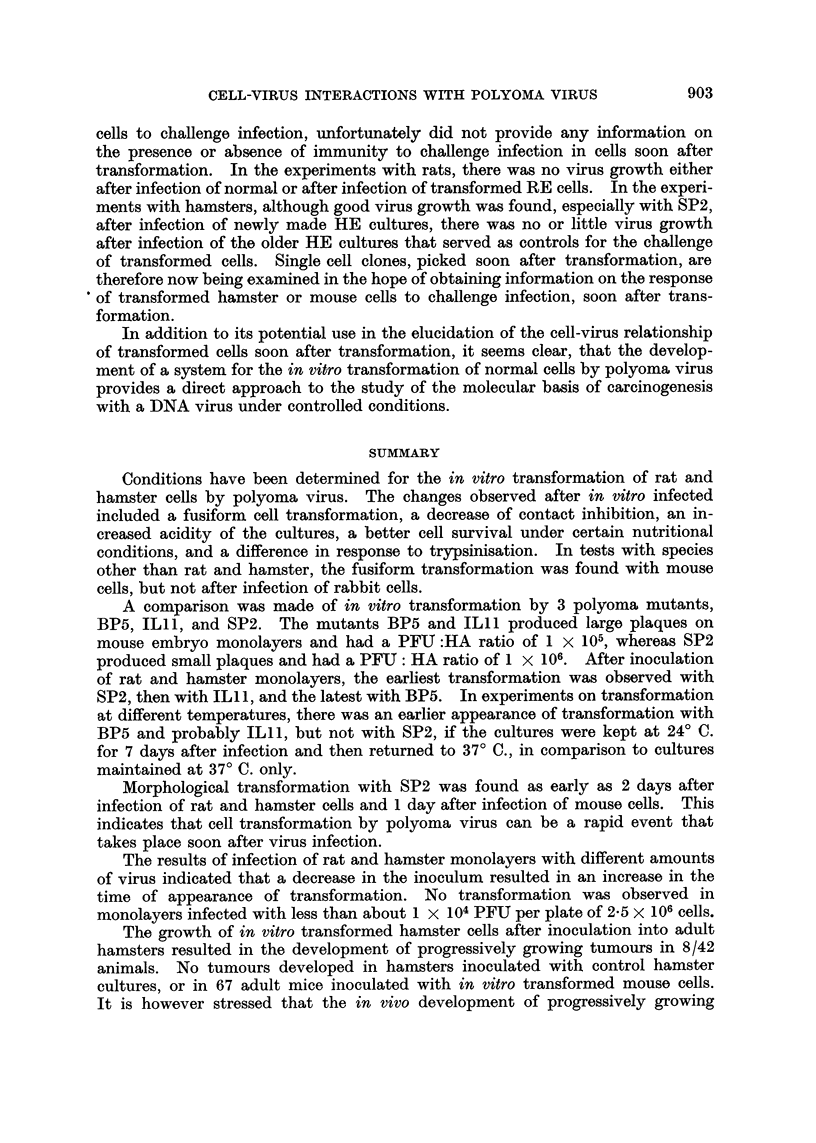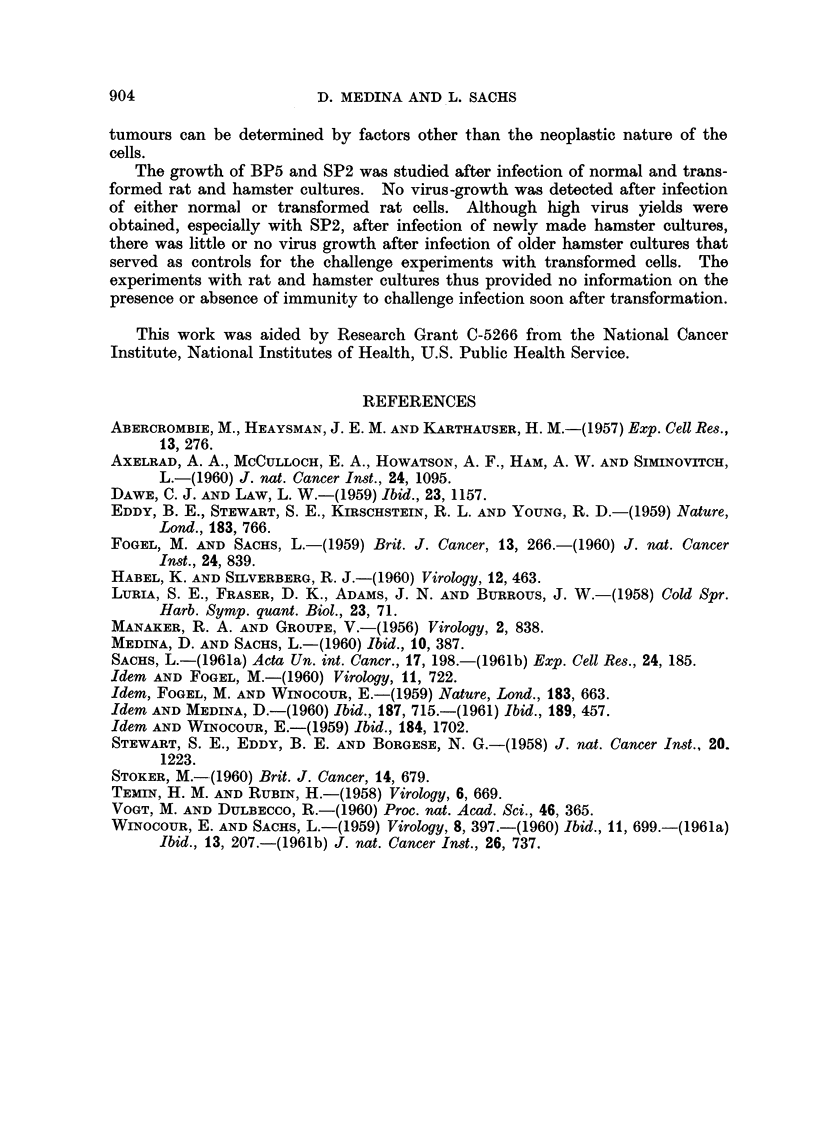# Cell-virus Interactions with the Polyoma Virus: The Induction of Cell Transformation and Malignancy in vitro

**DOI:** 10.1038/bjc.1961.102

**Published:** 1961-12

**Authors:** D. Medina, L. Sachs

## Abstract

**Images:**


### CELL-VIRUS INTERACTIONS WITH THE POLYOMA VIRUS: THE INDUCTION OF CELL TRANSFORMATION AND MALIGNANCY *IN VITRO*

D. MEDINA AND L. SACHS

*From the Laboratories of Virology and Genetics, Weizmann Institute of Science, Rehovoth, Israel*

Received for publication October 10, 1961

IN the previous studies with polyoma virus reported in this series, an analysis has been presented of the cell-cirus interactions in tumour cells induced *in vivo* (Sachs and Winocour, 1959; Sachs and Fogel, 1960; Winocour and Sachs, 1961*a*), and of the lytic interaction after virus infection *in vitro* (Winocour and Sachs, 1960). In addition to the production and study of the lytic interaction *in vitro*, it is, of course, also desirable to produce and analyse *in vitro* the non-lytic interaction that results in the change of a normal into a tumour cell. In parallel with these studies, experiments were therefore undertaken on the development of a system for the *in vitro* induction of cell transformation and malignancy and on the use of this system for the investigation of cell-virus relationships. Data on the development of such an *in vitro* system for embryo and kidney cells from mice, hamsters and rats have previously been reported (Medina and Sachs, 1960; Sachs and Medina, 1961). There have also been independent reports of *in vitro* transformation after infection of mouse and hamster cells (Dawe and Law 1959; Vogt and Dulbecco, 1960). The present paper is concerned with the requirements for *in vitro* cell transformation including the use of virus mutants, and with the malignant nature and cell-virus relationships of the *in vitro* transformed cells. Transformation will be referred to in this paper as a stable non-lytic change in the cell produced as the result of virus infection.

### MATERIALS AND METHODS

### *Culture media*

The two media used in the present experiments consisted of 0·5 per cent lactalbumin hydrolyzate in Earle's saline, and Eagle's medium with a four-fold concentration of amino and vitamins. These media will be referred to as LA and EM, respectively. The media were supplemented with either horse serum (HoS) or calf serum (CaS) as indicated in the text.

### *Tissue culture*

For the embryo tissue cultures, mouse embryos were excised at about the 15th day, rat embryos at about the 16th day, and hamster embryos at about the 11th day of gestation. Primary cultures were made with trypsin dispersed cell suspensions, prepared as described previously (Winocour and Sachs, 1960), at a seeding level of $8 \times 10^6$ cells with 10 ml. medium in 100 mm. petri dishes.

Secondary cultures were made after 4 to 5 days by seeding 2 to 2·8 × $10^6$ cells (depending on the experiment) with 4 ml. of medium in 50 mm. petri dishes. Unless otherwise stated, mouse and rat cultures were prepared in LA and 10 per cent HoS. However since LA with either HoS or CaS, or EM with HoS, did not give sufficiently good monolayers of hamster cells, hamster primary and secondary cultures were made in EM and 10 per cent CaS. Plastic petri dishes (Falcon Plastic Co., U.S.A.) were generally preferred to glass petri dishes for the rat and hamster cells, since they gave better cell growth.

Kidney cultures were seeded at 2 × $10^6$ cells in 50 mm. petri dishes. Those from young rats and hamsters were made with kidneys from 3 to 9 day old animals, and those from adults with kidneys from 2 to 3 months old animals. Hamster tumours induced *in vivo* were made into cell suspension as described previously (Sachs and Fogel, 1960), and seeded at 2 × $10^6$ cells in 50 mm. petri dishes with EM and 10 per cent CaS.

All cultures were incubated in a humidified incubator supplied with a constant flow of 10 per cent $CO_2$ in air. In each experiment, the results were based on the comparison of at least 4 experimental and 4 control petri dishes.

### *Virus mutants*

Three polyoma virus stocks originating from 3 sources have been used in these experiments, and virus from the 3 sources will be referred to as mutants IL11, BP5, and SP2. The IL11 virus, which could also be referred to as the wild type (Winocour and Sachs, 1959) was a re-isolation from a mouse parotid tumour induced by virus from 3919 (Stewart, Eddy and Borgese, 1958) obtained through the courtesy of Dr. S. E. Stewart and Dr. B. E. Eddy; BP5 was a plaque purified stock of a polyoma virus isolated in Toronto from a mouse mammary tumour, and kidney supplied by Dr. M. Stoker (Stoker, 1960); and SP2 was a small plaque mutant isolated after *in vitro* infection of L cells with a non-plaque purified stock of IL11 (Winocour and Sachs, 1961*b*). All virus stocks were made on mouse embryo monolayers, as for previous experiments (Winocour and Sachs, 1960, 1961*a*), from 3 times plaque purified virus.

### *Plaque assay, haemagglutination, and cell disruption for total virus*

The plaque assay was carried out as described previously (Winocour and Sachs, 1960) using mouse embryo secondary monolayers with LA and 15 per cent HoS. The mouse embryos were in all cases taken from the inbred mouse strain C57B1/6. When glass petri dishes were used, an initial agar nutrient overlay of 8 ml., followed by an overlay of 2·5 ml. containing neutral red on day 6, was sufficient to maintain the monolayers for 21 days or longer (Winocour and Sachs, 1960). However, in order to maintain monolayers longer than 14 days on plastic petri dishes, it was found necessary to use an initial agar nutrient overlay of 10 ml., followed by 3 ml. of overlay containing neutral red on day 6.

Haemagglutination tests were made with guinea-pig red blood cells as described previously (Fogel and Sachs, 1959). To counteract any possible haemagglutination inhibitors, samples were heated at 56° C. for 30 min. before they were tested.

Cell disruption for total virus was carried out by removing the cells from the petri dishes with a rubber wedge, and vibrating the cell suspension at full power for 3 minutes in a Raytheon 200-watt 10-kc magnetostrictive oscillator (Winocour and Sachs, 1960).

*Animals*

The mice used in the present experiments were from the inbred lines C57Bl/6, SWR, and DBA/2, whereas the rats (albino), and golden hamsters were from random bred colonies.

### EXPERIMENTAL

*Transformation of rat and hamster cells under different culture conditions*

In order to determine optimum conditions for *in vitro* transformation of rat and hamster cells, the first experiments were concerned with the changes produced after *in vitro* infection, and subsequent growth of the cultures, in different media and sera, and at different incubation temperatures. This original series of experiments was carried out with virus BP5, and some of the results have already been reported (Sachs and Medina, 1961). Monolayers were infected with 0·1 ml. of virus suspension at a virus cell ratio of 3 to 10 plaque forming units (PFU) per cell and with an adsorption period of 3 hr. Both rat and hamster primary and secondary embryo cultures, and primary kidney cultures, were used.

In the experiments with different media, 5 and 10 per cent serum was first tested, and 10 per cent was the then chosen in all further experiments. Rat cultures were tested in LA or EM with HoS or CaS, but since LA with either HoS or CaS was not an adequate medium for the growth of hamster cells, hamster monolayers were made in EM and CaS and then tested in EM and HoS or CaS. The experiments on different temperatures originated from the results on the effect of temperature on the frequency of lysogenisation with bacteriophage (Luria *et al.*, 1958), and it had been found in some of the original experiments that a much clearer transformation with BP5 could be obtained in cultures maintained at 24° C. for 5 to 8 days after infection, and then transferred back to 37° C., when compared to cultures kept at 37° C. only (Sachs and Medina, 1961). After growth of the monolayers at 37° C., most experimental groups therefore contained cultures maintained after infection either at 37° C. only, or at 24° C. for 5 to 8 days after infection.

The results of these experiments with BP5 are given in Table I. The data confirm those obtained previously (Sachs and Medina, 1961) in showing that *in vitro* transformation could be obtained in the appropriate conditions in all the tissues tested. The most suitable medium for transformation of each tissue, as established from these experiments, is also given in Table I. Secondary embryo cultures were more uniform in appearance than primaries, so that transformation could be more easily observed in secondaries. In the HE cultures, CaS tended to cause elongation of the cells after some days even in the controls. The use of EM and HoS after infection rather than EM and CaS, thus often produced a clearer early difference between the infected and control cultures. It should be mentioned that even with the most suitable medium, there was still occasionally some variation in the transformation observed, and the causes of this variation are being further studied. In all the cultures maintained at 24° C., there were only rarely slight changes at this temperature, and transformation was always obtained after the cultures were put back to 37° C. It should however be noted that transformation could also be obtained without the low temperature treatment.

TABLE I.—*Changes*[a] *Inducted by Polyoma Mutant BP5 After* in vitro *Infection of Rat and Hamster Cells*

| Tissue culture | Number of positive experiments[b] LA + HoS | LA + CaS | EM + HoS | EM + CaS | The most suitable medium chosen[c] |
|---|---|---|---|---|---|
| RE[d] primary . | 4/4[e] | 1/1 | 1/4 | 1/5 | LA + HoS |
| RE secondary . | 5/5[e] | 3/3 | 0/1 | NT | |
| RK young . . | 1/1 | NT | 0/1 | 0/1 | — |
| HE primary . | NT[f] | NT | 3/5 | 5/5 | EM + HoS or CaS |
| He secondary . | NT | NT | 3/4[e] | 1/2 | |
| HK young . . | NT | NT | 7/11[e] | 3/4 | EM + CaS |
| HK adult . . | NT | NT | 4/6 | 2/2 | |

[a] Including morphological transformation of the cells, acidity, and other changes mentioned in the text.
[b] Most of the groups with more than 1 experiment were tested at 24° C. → 37° C. and at 37° C. only. All cultures were infected at a virus : cell ratio of 3 to 10 PFU per cell.
[c] 5 and 10 per cent serum were tested, and 10 per cent was then thosen for all further experiments.
[d] RE = rat embryo. RK = rat kidney. HE = hamster embryo. HK = hamster kidney.
[e] These groups showed a much clearer transformation when kept after infection for 5 to 8 days at 24°C. and then transferred to 37° C.
[f] LA was found to be an unsuitable medium for the growth of hamster cells.

*The appearance of transformed rat and hamster cultures*

As in the previous experiments (Sachs and Medina, 1961), several differences were noted between the infected and the control cultures. The changes included, in the infected cultures, changes in cell morphology; what is presumably a decrease of contact inhibition; increased acidity; better survival; and a difficulty in dispersing the cells with trypsin. Experiments are now in progress to determine to what extent all or only some of these *in vitro* changes are associated with transformation to neoplasia.

The main change in cell morphology in RE and HE was the elongation of cells (fusiform transformation, Fig. 1). The cells first become starlike, and then more elongated, and this morphological change was seen more clearly in cultures without a frequent medium change.

Another change which was particularly clear in HK and HE cultures, and was also seen in the rat cultures (Fig 1), was a criss-cross type of growth, which will be referred to as a decrease of contact inhibition (Abercrombie, Heaysman and Karthawser, 1957).

A very common change in all cultures was a marked increase in acidity, so that the medium of the infected cultures was more acid than in the controls. After changing the medium, it could be shown that the transformed cultures showed an increased acidity in less than 24 hr. after the medium change, whereas the number of cells in the transformed and control cultures was the same.

---

EXPLANATION OF PLATE

FIG. 1.—Living rat embryo secondary cultures.

A = Control cultures.

B = Fusiform shape of cells and criss-cross growth at 12 days after *in vitro* infection with SP2 at a virus : cell ratio of 5 PFU per cell. Phase contrast. ×170.

1

Medina and Sachs.

A better survival of infected cultures was found in both RE and HE by keeping cultures sufficiently long without medium change, or under agar. Whereas the cells of control cultures degenerated, under these conditions, there was no such degeneration in the infected cultures. When old cultures started to peel off the plate, a much faster shrinkage was noted in the infected (and especially in the transformed) cultures, which formed a compact ball of tissue in contrast to the much looser shrinkage of the controls. It was also found that whereas control cultures could be readily trypsinised and formed cell suspensions in the usual way, transformed cultures treated with the same trypsin solution came off the glass with difficulty and did not form good cell suspensions.

RE monolayers infected with $1 \times 10^7$ PFU of the mutant SP2 (see next section) previously incubated with rabbit anti-polyoma antiserum for 30 min. showed no *in vitro* transformation.

There was no obvious cytopathic effect in any of the experiments shown in Table I, although a cytopathic effect was found after virus infection of HE with SP2. Some cell deaths seemed to occur in infected RE cultures, but the appearance of the spherical cells, free in the medium, was not the same as the clusters produced after the cytopathic effect in mouse and hamster cultures. Infected RE monolayers also often showed groups of small cells, or what may have been cell fragments, not found in the controls.

*Transformation with different virus mutants*

In a search for mutants with a better *in vitro* transforming activity than BP5, tests for transformation were made with the two other polyoma mutants IL11 and SP2. Of these 3 mutants, BP5 and IL11 produce large plaques and have a PFU : HA ratio of $1 \times 10^5$ (Winocour and Sachs, 1960), whereas SP2 produces small plaques and has a PFU : HA ratio of $1 \times 10^6$ (Table II). The

TABLE II.—*Plaque Size and Haemagglutination of* 3 *Polyoma Mutants*[a]

| Virus mutant | Virus origin[b] | Plaque size | PFU : HA ratio |
|---|---|---|---|
| SP2 | *In vitro* infected L cells | Small | $1 \times 10^6$ |
| IL11 | Mouse parotid tumour | Large | $1 \times 10^5$ |
| BP5 | Mouse mammary tumour | Large | $1 \times 10^5$ |

[a] The virus stocks compared in this table all had titers of $1 \times 10^8$ PFU per ml.
[b] For further details see Materials and Methods.

plaques of BP5 and IL11 when tested on mouse embryo monolayers in LA and 15 per cent HoS usually appear on day 7 or 8 and are about 12 mm. in diameter on day 20. Plaques of SP2, like those of SP1 (Medina and Sachs, 1960), usually appear 2 to 3 days later i.e. on day 10 or 11, show almost no increase in size, and are about 0·5 mm. in diameter on day 20. Neutralisation tests have shown that there is cross neutralisation between SP1, SP2, BP5 and IL11.

The comparative transformation tests with BP5, IL11, and SP2 were made with RE and HE secondaries inoculated at a virus : cell ratio of 3 to 10 PFU per cell. After the production of monolayers at 37° C., the cultures were infected and then grown either at 24° C. for 7 days and then transferred back to 37° C., or at 33° C. only, or at 37° C. only. The RE cultures were tested in LA and 10 per cent HoS, and the HE cultures in EM and 10 per cent HoS. In each experiment the 3 mutants were tested together on monolayers made from the same embryos prepared at the same time. The earliest appearance of morphological transformation was recorded, and the results are given in Table III. These data show that there was a difference in the earliest appearance of morphological transformation produced by the 3 mutants.

TABLE III.—*The Earliest Appearance of Transformation With Different Virus Mutants*

| Virus mutant | 24° → 37°[a] HE[b] | 33° RE | 33° HE | 37° RE | 37° HE | Average RE | Average HE |
|---|---|---|---|---|---|---|---|
| SP2 | 3[c] | 2 | 2–4 | 2 | 3 | 2 | 3 |
| IL11 | 3 | 4 | 9 | 4 | 4 | 4 | 5–6 |
| BP5 | 3 | 7 | 3–13 | 7 | 13 | 7 | 7–8 |

[a] Seven days at 24° after infection and then transferred to 37° C.
[b] HE = hamster embryo. RE = rat embryo.
[c] Day when transformation was first observed, not including days at 24° C. All cultures were infected at a virus : cell ratio of 3 to 10 PFU per cell.

It can be seen from these results, that in the 24° C. → 37° C. experiments all 3 mutants showed the earliest appearance of transformation 3 days after returning the cultures to 37° C. But in cultures kept at either 33° C. or 37° C, only, SP2 produced the earliest and BP5 the latest transformation. The mutants can thus be arranged according to the speed of transformation in mass cultures in the order SP2-IL11-BP5. The experiments with BP5 again show a generally earlier appearance of transformation in 24° C. → 37° C. than in 37° C. or 33° C. only, and the same also seems to apply to IL11. It is significant that an obvious transformation can be observed with SP2 as early as 2 days after infection, thus indicating that cell transformation by polyoma virus can be a rapid event that can occur soon after virus infection.

*The minimum virus inoculum required for transformation of rat and hamster cultures*

During the course of attempts to obtain discrete foci of transformed cells for quantitative studies and for the comparison of plaque forming units with transforming units, it was noted that there seemed to be a minimum virus inoculum below which no transformation was observed after infection of undisturbed cultures, i.e. cultures that had not been passaged after infection. Cultures containing $2{\cdot}5 \times 10^6$ RE or HE cells were therefore infected with different amounts of BP5 or SP2, in order to determine the time of transformation with different virus inputs and the minimum virus inoculum required for transformation. The results of such experiments at 24° C. → 37° C., and at 33° C., are given in Table IV.

The data show with both BP5 and SP2, that there was a considerable increase in time before transformation was observed as the virus : cell ratio of the inoculum decreased, and that no transformation was observed in any of the experiments with an input less than about $10^4$ PFU per culture. Since experiments number 164 and 141 were observed for 20 and 24 days respectively, this time would appear to have been sufficiently long to detect any cell transformation in the monolayer

TABLE IV.—*The Minimum Virus inoculum for Transformation in Mass Cultures*

| Experiment No. | Type of cell | Virus mutant | Temperature | Number PFU inoculated per plate[a] | Virus : cell ratio | Transformation first observed on day[b] | Duration of experiment (days) |
|---|---|---|---|---|---|---|---|
| 164 | HE[c] | BP5 | 24°→37°[d] | $5\times10^{6}$, $5\times10^{5}$ | 2 : 1, 1 : 5 | 2 | 20 |
| | HE | BP5 | 24°→37° | $5\times10^{4}$[e] | 1 : 50 | 13 | |
| 194 | HE | SP2 | 33° | $1\times10^{7}$ | 4 : 1 | 6 | 13 |
| | HE | SP2 | 33° | $1\times10^{6}$, $1\times10^{5}$ | 1 : 2·5, 1 : 25 | 10 | |
| 141 | RE | BP5 | 24°→37° | $1\times10^{7}$ | 4 : 1 | 1 | 24 |
| | RE | BP5 | 24°→37° | $1\times10^{6}$ | 1 : 2·5 | 8 | |
| | RE | BP5 | 24°→37° | $1\times10^{4}$[f] | 1 : 250 | 12 | |
| 191 | RE | SP2 | 33° | $1\times10^{7}$ | 4 : 1 | 2 | 12 |
| | RE | SP2 | 33° | $1\times10^{6}$ | 1 : 2·5 | 10 | |

[a] Lower inoculations of virus, which did not transform the cultures, do not appear in this table.
[b] Not including days at 24° C.
[c] HE = hamster embryo. RE = rat embryo.
[d] Seven days at 24° C. and then transferred to 37° C.
[e] Inoculum of 5 × $10^3$ PFU showed only acidity in the inoculated cultures.
[f] Inoculum of 1 × $10^5$ PFU was not tested in this experiment.

especially in the RE cultures where morphological transformation was readily observed. These results thus indicate that a minimum virus inoculum is required for cell transformation to be observed in the undisturbed infected rat and hamster cultures.

*Changes in tissues other than rat and hamster*

In addition to the experiments with rats and hamsters, cells from rabbit embryo, human amnion, monkey kidney, and mouse embryo, were tested for *in vitro* transformation either with SP2 or with all 3 mutants. The results of these tests are given in Table V.

In 13 experimental groups with rabbit embryo secondary cultures which included the use of different temperatures, the 3 virus mutants, and EM with either CaS or HoS, the only change observed was an increased acidity of the virus infected cultures in one experiment. The possible effect of LA could not be tested with rabbits, since it was found to be an unsuitable medium for the adequate growth of rabbit cells. In experiments with SP2 and growth at 37° C. no change was found after infection of human amnion primaries, whereas an increased acidity was noted after infection of monkey kidney primaries.

Infection of mouse embryo cultures with SP2 produced a rapid fusiform transformation which was observed as early as 1 day after infection in an experiment at 33° C., and 2 days after infection in another experiment at 37° C. (Table V). The early appearance of this transformation confirms the results with rat and hamster cells in showing that cell transformation can be a rapid event that takes place soon after virus infection. Fusiform transformation has also been found after infection of mouse embryo cells with IL11 and BP5, and after infection of mouse kidney cells (Sachs and Medina, 1961).

TABLE V.—*Changes After* in vitro *Infection of Cells Other than Hamster and Rat*

| Type of cell | Virus mutant | Medium | Temperature | Nature of change in infected culture | Changes first observed on day[c] | Duration of experiment (days) |
|---|---|---|---|---|---|---|
| Rabbit embryo | IL11 | EM+HoS or CaS | 24°→37°[a] | Acidity[b] | 3 | 15 |
| | | | 33° | No change | — | 15 |
| | | | 37° | ,, ,, | — | 15 |
| | BP5 | do. | 24°→37° | ,, ,, | — | 15 |
| | | | 33° | ,, ,, | — | 20 |
| | | | 37° | ,, ,, | — | 15 |
| | SP2 | do. | 33° | ,, ,, | — | 15 |
| | | | 37° | ,, ,, | — | 15 |
| Human amnion | SP2 | EM+HoS | 33° | ,, ,, | — | 14 |
| Monkey kidney | SP2 | EM+CaS | 33° | Acidity and swollen cells | 5 | 14 |
| Mouse embryo | SP2 | EM+CaS | 30° | Increased granulation | 8 | 15 |
| | | | 33° | Fusiform transformation | 1–3 | 11 |
| | | | 37° | ,, ,, | 2 | 6 |

[a] Seven days at 24° C. and then transferred to 37° C.
[b] This group was tested only in EM + CaS.
[c] Not including days at 24° C. All cultures were infected at a virus : cell ratio of 2 to 5 PFU per cell.

*Tumour formation* in vivo *by growth of* in vitro *transformed cells*

The experiments on *in vitro* cell transformation described above, have been combined, in the case of hamsters and mice, with tests for tumour formation *in vivo* by inoculating transformed cultures into adult animals. Adult animals were used in order to differentiate tumours produced by growth of transformed cells from those induced in the host by virus in the inoculum, and to avoid an early death of the host animals.

In the hamster experiments, including those reported earlier (Sachs and Medina, 1961), 66 animals, 1 to 8 months old, were inoculated with transformed or normal control hamster embryo cells. The cells were removed from the petri dishes by peeling off fragments of the cell layer with a rubber wedge, and each animal was injected subcutaneously in the middle of the back with 1 to 3 $\times$ $10^6$ cells in EM and 10 per cent HoS. The results (Table VI) show that tumours were formed in 8/42 hamsters inoculated with cells from transformed cultures, but that there were no tumours in 24 hamsters inoculated with cells from non-infected control cultures.

All the tumours in these experiments grew progressively, and were localised at the site of injection of the cells. They grew either under the skin or intradermally, and histologically they were classified as sarcomas. The intradermal neoplasms were very hard, and in addition to amorphic material, they included cartilage and bone, that had apparently differentiated from the embryonic cells. In only one animal (the tumour palpable after 42 days), was a tumour found in addition to that at the site of inoculation, and this animal died with a heart

TABLE VI.—*Tumour Formation in Adult Hamsters by Growth of in vitro Transformed Hamster Embryo Cells*

| Virus mutant | Temperature treatment of culture | Days after infection | Days after transformation | Number of animals with tumour / Number animals inoculated | Tumour first palpable (days after inoc.) | Observation period (months) |
|---|---|---|---|---|---|---|
| BP5 . | . 24°→37°[a] | 12, 38, 38 | 1, 28, 28 | 3/22 | 48, 37, 54 | 5–6 |
| | 33° | 17 | 14 | 0/4 | — | 5 |
| SP2 . | . 24°→37° | 12 | 1 | 1/4 | 42 | 5 |
| | 33° | 15, 17[b] | 11 | 4/7 | 52, 25, 129 | 5 |
| | 37° | 15 | 15 | 0/5 | — | 4 |
| Total . | — | — | — | 8/42 | — | — |
| Controls (non- | . 24°→37° | 12, 38 | — | 0/16 | — | 5–6 |
| | 33° | 15 | — | 0/5 | — | 4–5 |
| | 37° | 15 | — | 0/3 | — | 4–5 |
| Total . | — | — | — | 0/24 | — | — |

[a] Seven days at 24° C. and then transferred to 37° C.
[b] Tumours developed only in animals inoculated with transformed cells from the 15-day group.
[c] All cultures were infected at a virus : cell ratio of 5 PFU per cell.

tumour that may have been produced by virus in the inoculum. The time of appearance of palpable tumours in adult animals, the strict localisation of the tumour at the site of cell inoculation, and the existence of differentiated embryonic cells, all indicate that these tumours were produced by growth of transformed cells, and not induced in the host by virus in the inoculum. Even the 2 tumours that were first palpable at 129 days after cell inoculation into 8 months old hamsters, were strictly localised at the site of inoculation and there were no tumours at other sites in the animal. It is thus possible that these late tumours were also produced by growth of the *in vitro* transformed cells, and they have therefore been included in Table VI and VII.

Transplantability *in vivo* was tested in the case of the tumours palpable at 54, 52, and 25 days, and all three tumours gave progressively growing transplants after grafting into adult hamsters. The hard tumours became soft on the first passage.

It can be seen from Table VI that although tumours were produced after *in vitro* transformation with both BP5 and SP2, not all the inoculated transformed cultures resulted in the *in vivo* development of neoplasms. The *in vivo* growth of transformed cells did not seem to be influenced by the sex of the recipient, but the data in Table VII indicate, that the number of animals with neoplasms, and the time of appearance of palpable tumours, seemed to be influenced by the age of the host.

In tests with BP5 on tumour induction after subcutaneous inoculation of virus only into adult animals, no tumours were observed 3 months after inoculation of $1 \times 10^7$ PFU into 5 hamsters injected when 1 month old, or 6 months after inoculation of $3 \times 10^7$ PFU into 5 hamsters injected when 5 months old. In a test with IL11, subcutaneous tumours were found, in 2 out of 5 hamsters, 5 months after inoculation of $4 \times 10^6$ PFU into 2 months old animals. It is of

TABLE VII.—*The Incidence of* in vitro *Induced Hamster Tumours in Different age Groups*

| Age of animals at time of inoculation (months) | Number of animals with tumours: Early[a] appearing | Late[b] appearing | Number of animals inoculated |
|---|---|---|---|
| 1–1·5 | 6 | 0 | 31 |
| 4–6 | 0 | 0 | 7 |
| 8 | 0 | 2 | 4 |
| Total | 8[c] | | 42[d] |

[a] Tumours palpable between 25 to 54 days after inoculation.
[b] Tumours palpable only at 129 days after inoculation.
[c] 3 ♂ and 5 ♀.
[d] 16 ♂ and 26 ♀.

interest to note that in this particular case, in contrast to the 2 tumours that were first palpable at 129 days after inoculation of *in vitro* transformed cells, and where the tumour bearing animals were still alive 4 months later, the 2 hamsters with tumours after inoculation of virus only died 2 to 3 weeks after the subcutaneous tumours were first palpable, presumably due to tumours at other sites.

In the experiments with mice, 101 animals, 1 to 6 months old, belonging to the inbred strain C57Bl/6, SWR, and DBA/2, were injected subcutaneously with $1 \times 10^6$ cells per animal of transformed or normal control isologous mouse embryo cultures. Although some of the cultures gave palpable growths *in vivo* these growths in all cases regressed, and no progressive growth of the cells was found in any of the mice (Table VIII).

TABLE VIII.—*The Absence of Tumour Formation by Growth of* in vitro *Transformed Mouse Embryo Cells Inoculated into Isologous Adult Mice*[a]

| Mouse strain | Inoculated tissue | Temperature treatment of culture[b] | Inoculated days after infection | Number of animals inoculated | Progressively growing tumour | Observation period (months) |
|---|---|---|---|---|---|---|
| C57BI/6 | Transformed | 33° | 7 | 20 | 0 | 5 |
| | Normal control | 33° | 7 | 8 | 0 | 5 |
| | Transformed | 37° | 7 | 12 | 0 | 5 |
| | Normal control | 37° | 7 | 4 | 0 | 5 |
| SWR | Transformed | 33° | 7 | 12 | 0 | 5 |
| | Normal control | 33° | 7 | 12 | 0 | 5 |
| | Transformed | 37° | 7 | 20 | 0 | 5 |
| | Normal control | 37° | 7 | 8 | 0 | 5 |
| DBA/2 | Transformed | 33° | 9 | 3 | 0 | 3 |
| | Normal control | 33° | 9 | 2 | 0 | 3 |

[a] *In vitro* transformation by virus SP2.
[b] Most groups were grown both in LA and EM with HoS or CaS.
All cultures were infected at a virus : cell ratio of 5 PFU per cell.

It should however be noted, that it has been shown in all the 3 strains of mice used in these experiments (Sachs, 1961), that the transplantability of readily transplantable mouse tumours can be inhibited by inoculation of mice receiving the tumour grafts with polyoma virus. All the *in vitro* transformed cultures contained virus, so that the absence of tumour formation *in vivo* by growth of the transformed mouse cells, does not necessarily mean that the transformed cells were not malignant. Further experiments to elucidate this point are now in progress.

*Virus growth after infection of normal hamster cells*

With the development of a system for the *in vitro* transformation of normal cells with polyoma virus, it was obviously of interest to determine the response of transformed cells to challenge infection. The results of challenge infection with tumour cells induced *in vivo* have previously been reported (Winocour and Sachs, 1961*a*), but the advantage of using *in vitro* transformed cells would lie in the theoretical possibility to test the response to challenge infection soon after cell transformation. As a control to such challenge experiments with transformed hamster and rat cells, a study was first made of the growth of polyoma virus after infection of normal cells.

Virus growth on normal hamster cells was determined with BP5 and SP2. Secondary HE cultures grown in EM and 10 per cent CaS, and containing $2 \times 10^6$ cells at the time of infection, were inoculated at 1 to 2 days after seeding with 0·1 ml. of stock virus at a virus : cell ratio of 5 PFU per cell. After 3 hours adsorption, the plates were washed 3 times, and 4 ml. of EM and 10 per cent HoS added. Free virus (FV) and/or total virus (TV) was assayed at various times in the same way as in previous experiments (Winocour and Sachs, 1960, 1961*a*) and virus yields were determined from a pool of 2 plates for each point on the growth curve. Additional $NaHCO_3$ was added to the medium when infected cultures became too acid.

A comparison between FV and TV in a growth curve for BP5, in cultures maintained at 24° C. for 7 days after infection and then transferred to 37° C., is given in Fig. 2. There was no growth of the virus at 24° C., but a sharp rise between the 8th and 9th day. The difference between TV and FV on day 9 indicates that as in the case of mouse embryo cells (Sachs, Fogel and Winocour, 1959 ; Winocour and Sachs, 1960) there is a considerable amount of cell associated virus in virus yielding hamster cells.

In another experiment with BP5, a comparison was made between growth at 24° C. → 37° C. and growth at 37° C. only, on HE secondaries prepared from the same embryos at the same time. The results for TV are given in Fig. 3. The curve for 24° C. → 37° C. again shows no growth at 24° C. and a sharp rise between the 8th and 9th day, whereas cultures maintained at 37° C. only show a much lower rise, and only a total increase from $8 \times 10^3$ PFU to $1{\cdot}5 \times 10^5$ PFU during a period of 15 days.

The results for TV at 37° C. and 33° C. only, after infection with SP2 are given in Fig. 4. In this experiment, a cytopathic effect, in addition to transformation, was found on day 4 at both temperatures. Although the rise at 33° C. seems to be somewhat later than the rise at 37° C., at both temperatures the final yield with SP2 was as high as that found after infection of mouse embryo cells (Winocour and Sachs, 1960).

These growth curves on HE thus indicate that after infection with BP5 the total yield per culture was higher in the experiment at 24° C. → 37° C. than at 37° C. only, and that at the latter temperature the virus yield was relatively low ; and furthermore that the highest virus yields were obtained after infection with

SP2 and growth at either 33° C. or 37° C. only. These results also suggest that there is a difference in the growth of BP5 and SP2, and this is now being further studied.

In order to determine the percentage of virus producing cells, HE cultures were infected with SP2 at a virus : cell ratio of 5 PFU per cell, with an adsorption period of 3 hr. The cultures were then washed 3 times and the cells suspended by trypsinisation. The cells were then again washed, incubated for 15 min. with antipolyoma antiserum, diluted, and cell dilutions plated for an infective

FIG. 2.—Growth curve of polyoma mutant BP5 on hamster embryo cultures infected at a virus : cell ratio of 5 PFU per cell. The cultures were maintained for 7 days at 24° C. after infection, and then returned to 37° C. The medium was changed on day 7.
TV = Total virus. FV = Free virus.

centre assay on mouse embryo monolayers. The hamster cells were fixed to the assay plate by placing a thin layer of nutrient agar on top of the cell suspension before adding the final amount of agar nutrient overlay (Winocur and Sachs, 1960). Plaques were counted on day 21 of the plaque assay, and the number of virus producing cells was determined by the relationship between the number of cells plated and the number of plaques. The HE cultures were grown at 37° C. only, and the percentage of virus producing cells was tested at time O, i.e. after the adsorption period, and at 7 days after infection.

The results gave 11 per cent virus producing HE cells at time O, and 4 per cent at day 7 (transformation in this experiment was observed on day 5). There was thus in this experiment no increase, and possibly even a decrease, in virus producing cells after transformation. The value of 11 per cent virus producing cells at time O, after infection with a virus : cell ratio of 5 PFU per cell, is comparable to the value obtained after infection of mouse embryo cells with IL11 (Winocour and Sachs, 1960).

*Virus growth after infection of normal rat cells*

Virus growth in cultures of normal rat cells was also determined with BP5 and SP2, and experiments were made at 24° C. → 37° C. and at 37° C. only. RE

Fig. 3.—Growth curve (total virus) at 24° C. → 37° C., and at 37° C., of polyoma mutant BP5 on hamster embryo cultures infected at a virus : cell ratio of 5 PFU per cell. In the 24° C. → 37° C. curve the cultures were maintained for 7 days at 24° C. after infection and then returned to 37° C. Both curves were determined on cultures prepared from the same embryos at the same time. The medium was changed on day 7.

secondary cultures, taken at 1 to 2 days after seeding, were infected at a virus : cell ratio of 3 to 5 PFU per cell with an adsorption period of 3 hr. The cultures were then washed 3 times, 4 ml. of LA and 10 per cent HoS added, and TV content determined at various times. All the infected cultures in these experiments were transformed in the usual way. The results of 4 experiments, 2 with SP2 and 2 with BP5, each made with RE secondaries from a different batch of embryos, are given in Fig. 5. The data show that no virus growth was detected with either

BP5 or SP2 at 24° C. → 37° C. or at 37° C. only. There has also been no detectable virus growth after infection of RE with virus IL11 and growth at 37° C. (Sachs and Winocour, 1959 ; Sachs, 1961*a*).

*The response of transformed cells to challenge infection*

In order to determine the response of transformed cells to challenge infection, cultures of *in vitro* transformed cells, and of tumour cells induced *in vivo*, were challenged with polyoma virus and tested for TV content.

Fig. 4.—Growth curve (total virus) at 33° C., and at 37° C., of polyoma mutant SP2 on hamster embryo cultures infected at a virus : cell ratio of 5 PFU per cell. Both curves were determined on cultures prepared from the same embryos at the same time. The medium was changed on day 8 and day 13.

In the experiments with *in vitro* transformed HE cells, cultures were taken at 14, 19 and 24 days after infection (to ensure as complete a transformation as possible) and challenged, with their controls, with either BP5 or SP2 at a virus : cell ratio of 3 PFU per cell. The results of one such challenge experiment, after an original infection with SP2 and challenge with SP2 are shown in Fig. 6. In this experiment the cells were challenged 19 days after the original infection (15 days after the first appearance of transformation) and the cultures were kept for 10 days at 33° C. after the challenge inoculation. It has been shown (Fig 4), that the final yield with SP2 at 33° C. was the same as at 37° C., and the use of 33° C. had the advantage in that the cultures could be readily maintained for 10 days even without a medium change.

Fig. 5.—Growth curves (total virus) at 24° C. → 37° C., and at 37° C., of polyoma mutants BP5 and SP2 on rat embryo cultures infected at a virus : cell ratio of 3 to 5 PFU per cell. In the 24° C. → 37° C. curves the cultures were maintained for 7 days at 24° C. after infection, and the returned to 37° C. Each curve was determined on cultures made from different embryos. The medium was changed on day 6 for BP5 and day 10 for SP2.

It can be seen from Fig. 6, that even the control cultures of normal cells show only a rise from 6 × $10^5$ PFU to 5·3 × $10^6$ PFU during a period of 10 days, and this is much less than the increase found at this temperature after infection of newly made HE cultures with SP2 (Fig. 4). In addition the transformed and non-challenged cultures in this experiment still contained a very high virus titer, so that no increase in TV content was detectable after challenge (Fig. 6). In other challenge experiments on cultures with TV contents of $4 \times 10^4$ PFU or $3 \times 10^5$ PFU taken at 14 and 24 days after infection with BP5, and challenged with BP5 or SP2, there was no virus growth in the controls during a 4 day period at 37° C , and also no increased virus yield after challenge infection of transformed cultures

Cells from 4 hamster primary tumours induced *in vivo*, and from the 6th and 14th transplant generation of an *in vivo* transplanted polyoma induced hamster tumour, also gave no virus growth, during a 4 day period at 37° C., after infection with either BP5 or SP2. These tumours showed no infectious virus production, and tests for inhibitors made with cultures from 3 of the primary tumours by a plaque inhibition test (Winocour and Sachs, 1961*a*), were all negative.

FIG. 6.—Challenge infection (total virus) with polyoma mutant SP2 of hamster embryo cultures transformed by SP2. The controls were normal hamster embryo monolayers of the same age as the transformed cultures. Both the transformed and normal control cultures were infected at 19 days after the original infection, with a virus : cell ratio of 3 PFU per cell, and then maintained at 33° C. for 10 days without a medium change.

■ = Transformed not challenged.
● = Transformed challenged.
▲ = Normal control challenged.

Owing to the absence or very low amount of virus multiplication after infection of the normal older HE cultures, it was thus unfortunately not possible to obtain information on the existence of immunity to superinfection by challenge of mass cultures of transformed cells taken at these short intervals after transformation, or by challenge of cells from *in vivo* induced tumours. The absence of virus growth has also been noted after infection of another polyoma induced tumour transplanted *in vivo*, a culture from normal hamster tissue, (Habel and Silverberg, 1960), and other *in vitro* transformed HE cells observed for 7 days after challenge (Vogt and Dulbecco, 1960).

Experiments on challenge infection with BP5 and SP2 were made with *in vitro* transformed RE cultures, but in accordance with expectation from the results in Fig. 6, there was no virus growth either in the controls or in the challenged transformed cultures.

### DISCUSSION

The present experiments have extended our previous observations (Sachs and Medina, 1961), in defining some of the conditions required for the *in vitro* induction of cell transformation and malignancy by polyoma virus. The *in vitro* changes observed after infection of rat and hamster tissues included a fusiform transformation of the cells, and this serves as a useful mark for experimental studies. Other changes observed in transformed cultures included the *in vitro* development of a decrease of contact inhibition, an increased acidity of the infected cultures, a better survival of infected cells under certain nutritional conditions, and a difference in response to trypsinisation. The results with rats indicate that increased acidity associated with transformation does not seem to be due to virus multiplication, since acidity was found in transformed rat cultures without virus growth. The increased acidity in rats and hamsters could be explained in that the metabolism of the transformed cells results in an increased acid production, as in the case of cultures of tumour cells induced *in vivo*.

In tests with tissues other than hamster and rat, an *in vitro* fusiform transformation, similar to that observed with mouse kidney cells (Sachs and Medina, 1961), was found after infection of mouse embryo cells. But no morphological changes were detected after infection of rabbit embryo cells. The apparent lack of such changes in rabbit cells under conditions that produce clear transformation in rat, hamster and mouse cells, may be associated with the fact that the tumours induced *in vivo* by polyoma in rabbits are fibromas that regress (Eddy *et al.*, 1959 ; Fogel and Sachs, 1960) in contrast to the different types of malignant tumours induced in the other 3 species.

The conditions for *in vitro* transformation that have been studied, included the use of virus mutants and of different temperatures. Of the 3 mutants used, BP5 and IL11 produced large plaques on mouse embryo monolayers and have a PFU : HA ratio of $1 \times 10^5$, whereas SP2 produced small plaques and has a PFU : HA ratio of $1 \times 10^6$. Virus IL11 (Winocour and Sachs, 1959, 1961*b*) seems to differ *in vivo* from BP5, in that the latter produces in hamsters a very high incidence of early kidney tumours (Axelrad *et al.*, 1960 ; Stoker, 1960). A comparison of the earliest appearance of morphological transformation after *in vitro* infection of mass cultures of HE and RE cells, showed that this transformation was first observed with SP2, then with IL11, and last with BP5. In experiments with different temperatures there was also an earlier appearance of morphological transformation with BP5 and probably IL11, but not with SP2, if the cultures were kept at 24° C. for 7 days after infection and then returned to 37° C., in comparison to cultures maintained at 37° C. only. Although an earlier transformation, not counting the days at 24° C., was thus observed with some mutants in cultures maintained at 24° C., the low temperature treatment was not essential in order to obtain transformation.

These results thus indicate that there are polyoma mutants with different *in vitro* transforming abilities. These mutants can thus be used for the further elucidation of the process of cell transformation, and other polyoma mutants including SP1 (Medina and Sachs, 1960 ; Sachs and Medina, 1960) are now being tested. Experiments are also now in progress with single cell clones, in order to elucidate to what extent the difference in the time of appearance of transformation observed after infection of mass cultures is due to a difference in the number of initially transformed cells, and/or to a difference in the speed of transformation per cell, and whether the morphological transformation is necessarily associated with the decrease of contact inhibition. In addition to plaque size and PFU : HA ratio, a difference in cell transformation would appear to be another useful *in vitro* marker for studies on the genetics of polyoma virus.

It is of interest that morphological transformation with SP2 was observed as early as 2 days after infection of rat and hamster cells and 1 day after infection of mouse cells. These results therefore indicate that cell transformation by polyoma virus can be a rapid event that takes place soon after virus infection.

Experiments on the inoculation of RE and HE monolayers with different amounts of virus, showed that a decrease in the inoculum resulted in an increas in the time of appearance of transformation, and that no transformatiion was observed, even with SP2, when monolayers were incoulated with less than about $1 \times 10^4$ PFU per plate. Since these results may be explained in that only 1 cell in about $10^2$ was initially transformed after inoculation of the monolayer, and that in addition there is some kind of cell interaction in which a large excess of normal cells prevents cell transformation, this effect of size of inoculum is under further investigation.

Inoculation of transformed cell cultures into adult animals produced progressively growing tumours *in vivo* in 8/42 hamster, and in 0/67 mice. No tumours were formed in 24 hamsters and 34 mice inoculated with uninfected control cultures. The results with hamsters thus show that *in vitro* transformed hamster cells can grow progressively after inoculation into adult hamsters. The results however also show, that not all transformed cultures grew progressively in hamsters, and that none grew progressively in mice. It has been found in all the 3 strains of mice tested (Sachs, 1961*b*) that the growth of readily transplantable tumours can be inhibited by inoculation of the grafted animals with polyoma virus. This demonstrates that the ability of tumour cells to grow progressively after grafting into animals is not only determined by the neoplastic nature of the grafted cells.

All the transformed mouse cultures inoculated into mice contained virus in the inoculum, and this virus would have been sufficient to prevent the *in vivo* growth of tumour cells. The data on the development of hamster tumours in relation to the age of the animal, and the rapid appearance of palpable tumours after inoculation of 18 to 20 day old hamsters (Vogt and Dulbecco, 1960) suggests the possibility that there may be antigenic changes associated with cell transformation which could explain the absence of progressive growth of some cultures after inoculation into adult animals *in vivo*. Thus, although the ability to grow progressively after grafting into animals can be taken as a criterion for tumours, the lack of ability to grow progressively after grafting *in vivo* should not automatically disqualify the transformed cells that do not give successful grafts from inclusion into the category of neoplasms.

In addition to the criterion of progressive growth *in vivo*, it would thus seem desirable to develop criteria for the definition of neoplasia *in vitro*. A decrease of contact inhibition, which has also been observed after *in vitro* transformation with Rous virus (Manaker and Groupe, 1956 ; Temin and Rubin, 1958), and a better survival under certain nutritional conditions, would seem to be satisfactory candidates for inclusion into such a definition.

The experiments on the response of mass cultures of transformed RE and HE

cells to challenge infection, unfortunately did not provide any information on the presence or absence of immunity to challenge infection in cells soon after transformation. In the experiments with rats, there was no virus growth either after infection of normal or after infection of transformed RE cells. In the experiments with hamsters, although good virus growth was found, especially with SP2, after infection of newly made HE cultures, there was no or little virus growth after infection of the older HE cultures that served as controls for the challenge of transformed cells. Single cell clones, picked soon after transformation, are therefore now being examined in the hope of obtaining information on the response of transformed hamster or mouse cells to challenge infection, soon after transformation.

In addition to its potential use in the elucidation of the cell-virus relationship of transformed cells soon after transformation, it seems clear, that the development of a system for the *in vitro* transformation of normal cells by polyoma virus provides a direct approach to the study of the molecular basis of carcinogenesis with a DNA virus under controlled conditions.

### SUMMARY

Conditions have been determined for the *in vitro* transformation of rat and hamster cells by polyoma virus. The changes observed after *in vitro* infected included a fusiform cell transformation, a decrease of contact inhibition, an increased acidity of the cultures, a better cell survival under certain nutritional conditions, and a difference in response to trypsinisation. In tests with species other than rat and hamster, the fusiform transformation was found with mouse cells, but not after infection of rabbit cells.

A comparison was made of *in vitro* transformation by 3 polyoma mutants, BP5, IL11, and SP2. The mutants BP5 and IL11 produced large plaques on mouse embryo monolayers and had a PFU :HA ratio of $1 \times 10^5$, whereas SP2 produced small plaques and had a PFU : HA ratio of $1 \times 10^6$. After inoculation of rat and hamster monolayers, the earliest transformation was observed with SP2, then with IL11, and the latest with BP5. In experiments on transformation at different temperatures, there was an earlier appearance of transformation with BP5 and probably IL11, but not with SP2, if the cultures were kept at 24° C. for 7 days after infection and then returned to 37° C., in comparison to cultures maintained at 37° C. only.

Morphological transformation with SP2 was found as early as 2 days after infection of rat and hamster cells and 1 day after infection of mouse cells. This indicates that cell transformation by polyoma virus can be a rapid event that takes place soon after virus infection.

The results of infection of rat and hamster monolayers with different amounts of virus indicated that a decrease in the inoculum resulted in an increase in the time of appearance of transformation. No transformation was observed in monolayers infected with less than about $1 \times 10^4$ PFU per plate of $2{\cdot}5 \times 10^6$ cells.

The growth of *in vitro* transformed hamster cells after inoculation into adult hamsters resulted in the development of progressively growing tumours in 8/42 animals. No tumours developed in hamsters inoculated with control hamster cultures, or in 67 adult mice inoculated with *in vitro* transformed mouse cells. It is however stressed that the *in vivo* development of progressively growing tumours can be determined by factors other than the neoplastic nature of the cells.

The growth of BP5 and SP2 was studied after infection of normal and transformed rat and hamster cultures. No virus-growth was detected after infection of either normal or transformed rat cells. Although high virus yields were obtained, especially with SP2, after infection of newly made hamster cultures, there was little or no virus growth after infection of older hamster cultures that served as controls for the challenge experiments with transformed cells. The experiments with rat and hamster cultures thus provided no information on the presence or absence of immunity to challenge infection soon after transformation.

This work was aided by Research Grant C-5266 from the National Cancer Institute, National Institutes of Health, U.S. Public Health Service.

### REFERENCES

Abercrombie, M., Heaysman, J. E. M. and Karthauser, H. M.—(1957) *Exp. Cell Res.*, **13**, 276.

Axelrad, A. A., McCulloch, E. A., Howatson, A. F., Ham, A. W. and Siminovitch, L.—(1960) *J. nat. Cancer Inst.*, **24**, 1095.

Dawe, C. J. and Law, L. W.—(1959) *Ibid.*, **23**, 1157.

Eddy, B. E., Stewart, S. E., Kirschstein, R. L. and Young, R. D.—(1959) *Nature, Lond.*, **183**, 766.

Fogel, M. and Sachs, L.—(1959) *Brit. J. Cancer*, **13**, 266.—(1960) *J. nat. Cancer Inst.*, **24**, 839.

Habel, K. and Silverberg, R. J.—(1960) *Virology*, **12**, 463.

Luria, S. E., Fraser, D. K., Adams, J. N. and Burrous, J. W.—(1958) *Cold Spr. Harb. Symp. quant. Biol.*, **23**, 71.

Manaker, R. A. and Groupe, V.—(1956) *Virology*, **2**, 838.

Medina, D. and Sachs, L.—(1960) *Ibid.*, **10**, 387.

Sachs, L.—(1961a) *Acta Un. int. Cancr.*, **17**, 198.—(1961b) *Exp. Cell Res.*, **24**, 185.

*Idem* and Fogel, M.—(1960) *Virology*, **11**, 722.

*Idem*, Fogel, M. and Winocour, E.—(1959) *Nature, Lond.*, **183**, 663.

*Idem* and Medina, D.—(1960) *Ibid.*, **187**, 715.—(1961) *Ibid.*, **189**, 457.

*Idem* and Winocour, E.—(1959) *Ibid.*, **184**, 1702.

Stewart, S. E., Eddy, B. E. and Borgese, N. G.—(1958) *J. nat. Cancer Inst.*, **20**. 1223.

Stoker, M.—(1960) *Brit. J. Cancer*, **14**, 679.

Temin, H. M. and Rubin, H.—(1958) *Virology*, **6**, 669.

Vogt, M. and Dulbecco, R.—(1960) *Proc. nat. Acad. Sci.*, **46**, 365.

Winocour, E. and Sachs, L.—(1959) *Virology*, **8**, 397.—(1960) *Ibid.*, **11**, 699.—(1961a) *Ibid.*, **13**, 207.—(1961b) *J. nat. Cancer Inst.*, **26**, 737.